# Apoptotic cells promote circulating tumor cell survival and metastasis

**DOI:** 10.1038/s42003-025-08541-7

**Published:** 2025-07-29

**Authors:** Cassidy E. Hagan, Valerie M. Sheehan, Charles M. Rezanka, Meijie A. Li, Laura Martínez-Escardó, Kirsteen J. Campbell, Annelise G. Snyder, Mark Headley, Andrew Oberst

**Affiliations:** 1 Department of Immunology, University of Washington, Seattle, WA USA; 2 CRUK Scotland Institute, Glasgow, UK; 3 Department of Biology, Western Washington University, Bellingham, WA USA; 4 Translational Science and Therapeutics Division, Fred Hutchinson Cancer Research Center, Seattle, WA USA

**Keywords:** Metastasis, Cell death

## Abstract

During tumor progression and especially following cytotoxic therapy, cell death of both tumor and stromal cells is widespread. Despite clinical observations that high levels of apoptotic cells correlate with poorer patient outcomes, the physiological effects of dying cells on tumor progression remain incompletely understood. Here, we report that circulating apoptotic cells robustly enhance tumor cell metastasis to the lungs. Using intravenous metastasis models, we observed that the presence of apoptotic cells, but not cells dying by other mechanisms, supports circulating tumor cell (CTC) survival following arrest in the lung vasculature. Apoptotic cells promote CTC survival by recruiting platelets to the forming metastatic niche. Apoptotic cells externalize the phospholipid phosphatidylserine to the outer leaflet of the plasma membrane, which we found increased the activity of the coagulation initiator Tissue Factor, thereby triggering the formation of platelet clots that protect proximal CTCs. Inhibiting the ability of apoptotic cells to induce coagulation by knocking out Tissue Factor, blocking phosphatidylserine, or administering the anticoagulant heparin abrogated the pro-metastatic effect of apoptotic cells. This work demonstrates a previously unappreciated role for apoptotic cells in facilitating metastasis by establishing CTC-supportive emboli, and suggests points of intervention that may reduce the pro-metastatic effect of apoptotic cells.

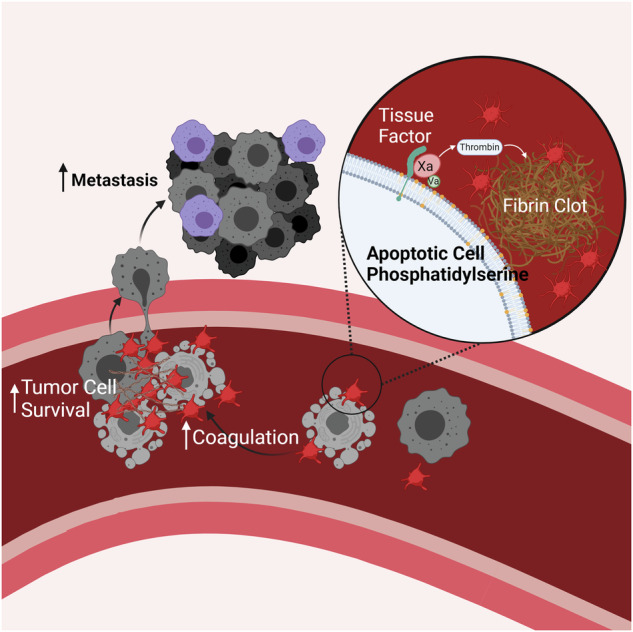

## Abstract (web summary)

Apoptotic cells promote metastasis through Tissue Factor and phosphatidylserine dependent procoagulant activity, establishing platelet-tumor cell niches which support tumor cell survival in circulation.

## Introduction

Metastatic disease, caused by the dissemination of tumor cells from a primary tumor to distant tissues, is the major cause of mortality in cancer patients^1^. Efforts to understand the process of metastasis have identified key features of circulating tumor cells (CTCs) that successfully seed distant tissues and defined aspects of the microenvironment required to support CTCs throughout the metastatic cascade^2^. CTCs must create a supportive niche to allow their survival in the hostile circulatory environment and upon arrest at distant tissue sites. A key feature of this process is the activation of coagulation by CTCs; this leads to the coating of CTCs by activated platelets, which provide survival signals and protect CTCs from shear stress and natural killer (NK) cell recognition^3–9^. Platelet activation is required for the subsequent recruitment of neutrophils and monocytes^10,11^ and increases endothelial permeability to support extravasation^12^. Further underscoring the importance of cell-cell interactions, CTC clusters have significantly enhanced metastatic potential when compared to individual CTCs^13–16^.

Overall, the metastatic process is extremely inefficient. Experiments tracking the fate of CTCs have found that <1% of cells survive the stresses associated with vascular dissemination^17,18^. Recognition by immune cells as well as shear stress leads to the demise of a majority of CTCs before they can arrest, extravasate, and form a metastatic tumor. While most research has focused on characteristics that allow rare CTCs to successfully metastasize, very little is understood about the effects of the greater portion of tumor cells that die during the process of metastasis.

Studies examining CTCs have found that a large proportion show molecular features of apoptosis, and in patients with breast cancer, the presence of apoptotic CTCs is correlated with progression to metastatic disease^19–23^. Furthermore, apoptosis within primary tumors has been shown to promote tumor growth via multiple mechanisms, including promoting selection for aggressive cellular clones^24^, releasing proliferative signals^25^, and recruiting and polarizing macrophage populations^26,27^. However, experimental evidence evaluating the effect of circulating apoptotic cells on metastasis is lacking.

Apoptosis is executed following the activation of caspases, a family of protease enzymes whose targets define the morphological changes associated with apoptotic cell death. Caspase-8 is activated following extrinsic stimuli, while caspase-9 is activated downstream of mitochondrial permeabilization during intrinsic apoptosis. Each pathway converges on the activation of executioner caspases 3 and 7, which are responsible for the highly ordered demise of the cell. Numerous caspase targets exist, resulting in canonical features of apoptosis such as cell shrinkage, membrane blebbing, externalization of phosphatidylserine from the inner to the outer leaflet of the plasma membrane, DNA degradation, and chromatin condensation. Apoptosis is considered an anti-inflammatory form of programmed cell death, inducing programs of tolerance and wound healing, consistent with its putative role in promoting tumor growth^28–30^.

Here, we show that apoptotic cells in circulation potently enhance metastasis by promoting the survival of CTCs. This effect requires the physical association of apoptotic cells with CTCs at sites of metastatic colonization, and is driven by the promotion of coagulation and platelet aggregation by apoptotic cells. The procoagulant activity of apoptotic cells is dictated by Tissue Factor (TF) activation, which occurs upon phosphatidylserine externalization on the apoptotic cell surface. The ability of apoptotic cells to promote metastasis correlates with the colocalization of tumor cells and apoptotic cells within platelet clots, establishing apoptotic cells as an important contributor to the early metastatic microenvironment supporting tumor cell survival in circulation.

## Results

### Circulating apoptotic cells increase lung metastasis in I.V. metastasis models

**Fig. 1:**
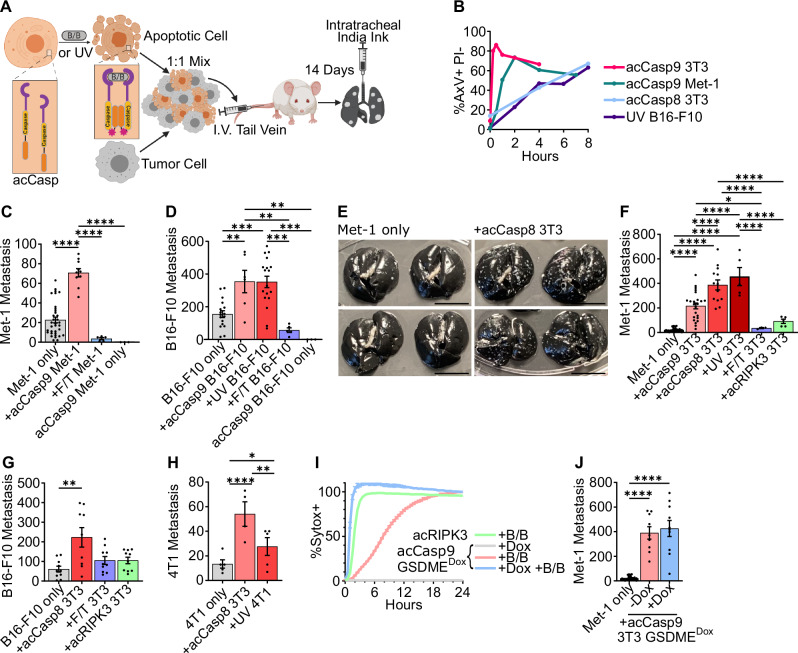
Circulating apoptotic cells increase lung metastasis in I.V. metastasis models. acCasp8, acCasp9, or acRIPK3 expressing cells were activated by treatment with B/B, UV irradiated, or killed by Freeze/Thaw lysis (F/T). Dying cells were mixed 1:1 with tumor cells and injected I.V., 14 days later, surface lung metastasis was quantified (**A**, **C**–**H**, **J**). Phosphatidylserine externalization and membrane integrity on cells after B/B or UV exposure were measured with Annexin V (AxV) and propidium iodide (PI) by flow cytometry (**B**). GSDME expression in acCasp9 3T3 GSDME^Dox^ cells was induced by doxycycline treatment (+Dox), followed by treatment with B/B to activate acCasp9 (**I**, **J**). Membrane permeability was measured by Sytox Green uptake (**I**). Scale bar is ~1 cm (**E**). Dots are biological replicates, Error bars represent SEM, statistical testing is Ordinary one-way ANOVA with Tukey’s multiple comparisons test (**C**–**J**). *Z*-scores from three replicate experiments were used for statistical testing (**H**, see supplementary Fig. 2). Diagram created in BioRender. Oberst, A. (2025) https://BioRender.com/ddyc016 (**A**).

Given the clinical correlation between apoptotic CTCs and progression to metastasis in patients with breast cancer^19,20^, we sought to design a system in which we could directly assess the impact of apoptotic cells during hematogenous dissemination of breast cancer. To do this, Met-1 cells, a mammary tumor cell line derived from MMTV-PyMT transgenic mice^31^, were injected intravenously (I.V.) into syngeneic (FVBN/J) immunocompetent mice at a 1:1 ratio with apoptotic cells (Fig. 1A). Apoptotic cells were prepared from cells lines expressing caspase-8 or caspase-9 fused to activatable FKBP^F36V^ dimerization domains (hereafter acCasp8, acCasp9). Incubating these cells with the nontoxic ligand B/B, a synthetic bivalent homolog of rapamycin, enforces caspase dimerization and activation. This system, which we and others have used in the past, gives us precise control over the activation of apoptosis^32–35^. After a short pulse incubation with B/B, ex vivo cells are committed to undergo apoptosis and are immediately processed for I.V. injections such that apoptotic cells undergo the apoptotic cascade in vivo. Inducing apoptosis through acCasp9 resulted in rapid externalization of phosphatidylserine (PS) while acCasp8 activation or UV irradiation resulted in slower, more heterogeneous apoptotic cell death (Fig. 1B). In all cases, induction of apoptosis led to 100% cell death as measured by membrane permeability (Supplementary Fig. 1). Fourteen days following I.V. injection of Met-1 cells alone or Met-1 cells mixed with dying cells, mice were euthanized and the number of metastatic nodules on the lung surface was quantified.

We observed that coinjection of apoptotic Met-1 cells with viable Met-1 cells increased the metastatic burden by approximately three-fold, while necrotic Met-1 cells killed via freeze/thaw (F/T) lysis^36^ had no impact on metastasis (Fig. 1C). We also examined a model of melanoma metastasis, B16-F10 cells injected I.V. into B6/J mice, and found a similar increase in metastasis with coinjection of apoptotic but not necrotic tumor cells (Fig. 1D). Induction of apoptosis in B16-F10 cells through both UV irradiation and acCasp9 activation had similar effects. We confirmed that apoptotic cells themselves could not form metastatic nodules; injecting only apoptotic cells resulted in no detectable tumor foci in the lung (Fig. 1C, D). To better model metastatic seeding, which occurs over time from a primary tumor, we implanted B6/J mice with B16.F10 flank tumors and subsequently performed multiple I.V. injections of B16.F10 cells alone or mixed with UV-irradiated B16.F10 tumor cells. We found that a similar phenotype exists in tumor-bearing mice; apoptotic tumor cells increase metastasis (Supplementary Fig. 2A, B).

CTCs can also be shed with stromal cells^37,38^. To examine whether apoptotic cells of non-tumor origin could also potentiate metastasis, we tested the effect of coinjecting apoptotic NIH-3T3 fibroblasts (3T3) in Met-1 and B16-F10 metastasis models. Strikingly, apoptotic 3T3s enhanced Met-1 metastasis by ~10–20 fold depending on the mechanism through which apoptosis was induced (Fig. 1E, F). Apoptotic 3T3s had a similar effect on B16-F10 metastasis as apoptotic B16-F10 cells (Fig. 1G). 4T1 cells, another common breast cancer cell line, showed a similar phenotype to Met-1 cells; coinjecting apoptotic 3T3 cells resulted in a robust increase in 4T1 metastasis, whereas apoptotic 4T1 cells resulted in a smaller increase in tumor cell metastasis (Fig. 1H, Supplementary Fig. 2C–F).

We confirmed that the allogenicity of 3T3s was not a relevant variable by observing that autologous mouse embryonic fibroblasts (MEF) derived from B6/J or FVBN/J embryos and then stimulated to undergo apoptosis with UV irradiation also enhanced Met-1 and B16-F10 metastasis to a similar degree as apoptotic 3T3s (Supplementary Fig. 2G, H). Our finding that allogeneic and autologous apoptotic fibroblasts, as well as apoptotic tumor cells, enhance metastasis suggests that the promotion of metastasis is a general feature of apoptotic cells, regardless of their origin or the mechanism of apoptosis induction.

As observed with necrotic Met-1 and B16-F10, necrotic 3T3 fibroblasts had no effect on metastasis in either model. Inducing necroptosis, a form of programmed lytic cell death, through acRIPK3 oligomerization in 3T3 cells^32,35^ also had no impact on metastasis (Fig. 1F, G). The caspase-3 cleavable pore-forming protein Gasdermin E (GSDME) is responsible for secondary necrosis following apoptosis^39^. Doxycycline (Dox) controlled overexpression of GSDME, causing cells to progress to secondary necrosis rapidly upon caspase activation, comparable to the rate of lysis after acRIPK3 oligomerization (Fig. 1I). However, GSDME expression did not impact the ability of acCasp9 cells to enhance metastasis. Together, this suggests that caspase-independent lytic cell death does not promote metastasis; however, cell lysis following caspase activation does not terminate the prometastatic effect of apoptotic cells (Fig. 1J).

### Apoptotic cells provide a survival advantage to circulating tumor cells

**Fig. 2:**
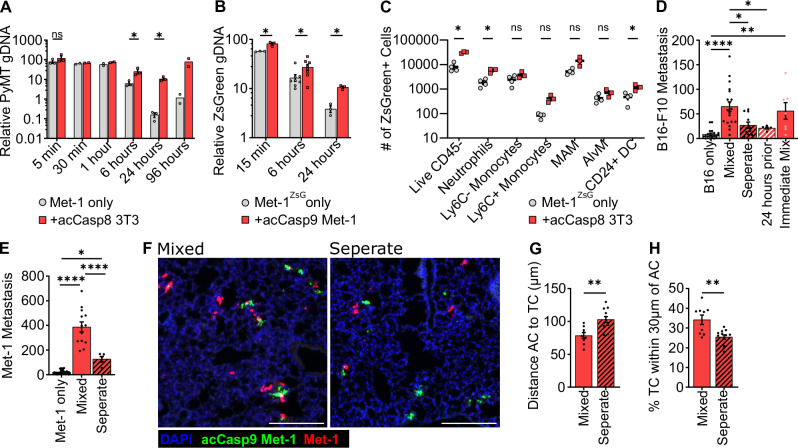
Spatial association of apoptotic cells and tumor cells provides an early survival advantage to tumor cells. Met-1 cells were injected I.V. with acCasp8 3T3 (**A**, **C**, **E**) or acCasp9 Met-1 (**B**, **F**–**H**). Lung gDNA was harvested at the indicated timepoints for qPCR (**A**, **B**) or lungs processed for FACS at 24 h (**C**). Met-1 cells expressed ZsGreen (Met-1^ZsG^) (**B**, **C**). B16-F10 cells were injected I.V. with acCasp8 3T3 (**D**). Tumor cells and apoptotic cells were mixed up to 1 h in advance (mixed) or immediately prior and injected in a single bolus, or injected in two sequential injections into opposite tail veins (separate), or apoptotic cells were injected 24 h prior to tumor cell challenge (**D**–**H**). Surface lung metastasis was quantified after 14 days (**D**, **E**). Lungs were harvested 1 h after I.V. for visualization by fluorescent microscopy, acCasp9 Met-1 cells were stained with CFSE (green) and Met-1 cells expressed mCherry (red), scale bar = 200 µm (**F**–**H**). Error bars represent SEM, Dots are biological replicates (**A**–**E**) or represent distinct slices of lung tissue taken from *n* = 3–4 biological replicates (**G**, **H**), statistical testing is unpaired *t*-test (**A**–**C**, **G**, **H**) or Ordinary one-way ANOVA with Tukey’s multiple comparisons test (**D**, **E**) AC apoptotic cell, TC tumor cell.

We next sought to assess when, during the dynamic process of tumor cell arrest, survival, and extravasation, apoptotic cells exert their prometastatic effect. To quantify tumor cell seeding and subsequent survival, we used qPCR to measure tumor-specific genomic DNA (PyMT or ZsGreen transgene) in the lung at timepoints from 5 min to 96 h post I.V. injection. Maximum tumor cell quantity in the lung was observed as early as 5 min following I.V. injection, indicating rapid arrest of tumor cells in the lung vasculature. The relative quantity of tumor cells declined exponentially over the next 24 h as previously reported^18^ (Fig. 2A, B). We concurrently quantified apoptotic cell genomic DNA in the lung and, as expected, apoptotic cells showed a similar logarithmic decline in abundance over 24 h, concomitant with their uptake by CD45+ phagocytes (Supplementary Fig. 3A–C). We found that tumor cell survival (as measured by persistence of tumor genomic material over the first 24 h) was increased in the presence of apoptotic cells. Coinjection of Met-1 tumor cells with apoptotic 3T3 cells resulted in an increase in PyMT gDNA (Met-1 cells) of ~4-fold at 6 h post-injection and 50-fold at 24 h post-injection (Fig. 2A). The sensitivity of this assay also allowed us to inject 10-fold fewer tumor and apoptotic cells to demonstrate that the effects of apoptotic cells persist even at lower concentrations in the blood (Supplementary Fig. 3D). Met-1 cells expressing ZsGreen to distinguish them from coinjected apoptotic acCasp9 Met-1 cells also showed increased gDNA persistence in the lungs at 6 and 24 h post-injection relative to Met-1 cells injected alone (Fig. 2B).

The exponential decline in tumor cell persistence over the first 24 h after I.V. injection is likely a result of those cells undergoing apoptosis themselves. At 1 h post-injection, we examined tumor cells for signs of active apoptosis. We observed that an average of 70% of tumor cells were positive for cleaved caspase-3 (CC3) and 40–60% of tumor cells externalized phosphatidylserine (PS) (Supplementary Fig. 3E–G). This suggests that a vast majority of tumor cells themselves are undergoing apoptosis. We observed a reduction in the fraction of PS+ tumor cells at 1-h post-injection when they were coinjected with apoptotic cells (Supplementary Fig. 3H). Together, these data suggest that apoptotic cells improve CTC survival by protecting tumor cells during this critical window of lung seeding.

We further characterized the fate of ZsGreen-expressing tumor cells using flow cytometry. The increased persistence of tumor gDNA when coinjected with apoptotic cells was mirrored in the increased number of live, unengulfed tumor cells based on FACS analysis (Fig. 2C, Supplementary Fig. 4A–C). Examining phagocytes that took up tumor cell-derived material revealed that tumor cells are phagocytosed primarily by non-alveolar macrophages and Ly6C- monocytes, as reported previously^40^, and there was no defect in the uptake of tumor cells in the presence of apoptotic cells (Fig. 2C, Supplementary Fig. 4B, C). We also did not observe any difference in the number of lung metastasis between WT and transgenic “BELMO” mice, which have enhanced phagocytic capacity as previously described^41^ (Supplementary Fig. 4D). These data indicate that apoptotic cells increase the survival of tumor cells within the first 24 h after injection, and do not act by limiting the uptake of tumor cells by phagocytes.

We then assessed the role of various immune cell types in controlling the promotion of metastasis by apoptotic cells, including adaptive immune cells, NK cells, and myeloid cells. Apoptotic cells retained their ability to promote metastasis in *Rag2*^*−/−*^ mice, which lack T and B cells, indicating that the adaptive immune compartment was not a primary driver of the effects of apoptotic cells (Supplementary Fig. 5A). Next, to examine the contribution of NK cells which play an important role in restricting CTC survival^42,43^, we administered an NK depletion antibody (anti-NK1.1) 72 and 24 h prior to I.V. metastasis challenge. Although depleting NK cells resulted in substantially higher metastatic burden, as previously reported^44^, apoptotic cells still provided a metastatic advantage, suggesting that apoptotic cells are acting via a mechanism other than protecting tumor cells from NK cell killing (Supplementary Fig. 5B, C). We interrogated the role of phagocytic myeloid cells by systemic depletion using clodronate liposomes. We confirmed depletion of myeloid cell subsets in the lungs using flow cytometry (Supplementary Fig. 6A, B). We observed no difference in the effect of apoptotic cells in the B16.F10 model after depleting macrophages with clodronate liposomes (Supplementary Fig. 6C). Interestingly, we did see a reduction in the effect of apoptotic cells on metastasis in the Met-1 model at 14 days and 24 h, consistent with previous reports of macrophages having an important role during the extravasation and growth phase of metastasis in this cell line^18^. However, there was no impact of clodronate liposomes on the enhanced survival of Met-1 at 6 h post-injection, suggesting that the earliest effects of apoptotic cells in promoting metastasis are independent of macrophages (Supplementary Fig. 6D–F). We also did not observe any differences in recruitment of various myeloid populations in the presence of apoptotic or necroptotic cells, and we found that the same myeloid populations phagocytosed Met-1, acCasp9 3T3, and acRIPK3 3T3 (Supplementary Fig. 6G, H).

We next sought to address whether temporal or spatial separation of apoptotic cells from tumor cells affected metastatic burden by injecting apoptotic cells 24 h prior to tumor cell challenge, or sequentially into the opposite tail vein from tumor cells. Pre-injecting apoptotic cells 24 h prior had no effect on metastatic burden in the lung, suggesting that apoptotic cells do not have long-lasting effects that impact CTC survival (Fig. 2D). Injecting apoptotic cells and tumor cells within 1 min of each other, but into opposite tail veins, also abrogated the prometastatic effect of apoptotic cells (Fig. 2D, E). This suggests that apoptotic cells do not promote metastasis systemically, but rather spatial or temporal association between apoptotic cells and tumor cells may be necessary for their prometastatic effect.

We considered the possibility that tumor cells might receive pro-survival signals from apoptotic cells during the co-incubation period prior to injection. However, we found that the duration of ex vivo contact between apoptotic cells and tumor cells did not influence their capacity to enhance metastasis. Combining apoptotic and tumor cells immediately prior to injection or up to 1 h in advance resulted in comparable enhancement of metastasis by apoptotic cells, suggesting that mixing tumor cells and apoptotic cells does not result in prometastatic ex vivo cell-cell signaling interactions (Fig. 2D).

Given that the murine lung contains over 1 km of total vasculature^45^, we wondered whether injecting apoptotic cells and tumor cells in separate veins reduced their spatial proximity within the lung. When injected together, apoptotic cells were often localized in close proximity to tumor cells within the lung (Fig. 2F). When injected separately into opposite tail veins, we observed a reduced percentage of tumor cells in very close proximity to apoptotic cells and an increased average distance between them (Fig. 2F–H). Bivariate Ripley’s K and nearest neighbor distance analyses demonstrated that there is a more pronounced spatial association between apoptotic cells and tumor cells when injected together vs. separately, and this association is higher than what would be expected from random distribution (Supplementary Fig. 7A–G). Injecting tumor cells and apoptotic cells in separate tail veins rather than mixed together did not impact the number of tumor or apoptotic cells that seeded the lung. (Supplementary Fig. 7H, I). These data indicate that apoptotic cells must be spatially associated with viable tumor cells in order to have a prometastatic effect. Notably, this association would occur naturally in the context of tumor cell clusters, which have been shown to have a metastatic advantage relative to single tumor cells^14,15,37,46^.

Together, these findings indicate that apoptotic cells promote tumor cell survival following initial seeding of the lung vasculature, and suggest that close spatial association between tumor cells and apoptotic cells in vivo is required for apoptotic cells to exert these effects. The spatial and temporal dynamics of apoptotic cell enhancement of metastasis led us to hypothesize that apoptotic cells were helping to establish an early protective microenvironment around CTCs.

### Apoptotic cells promote platelet aggregation on tumor cells

**Fig. 3:**
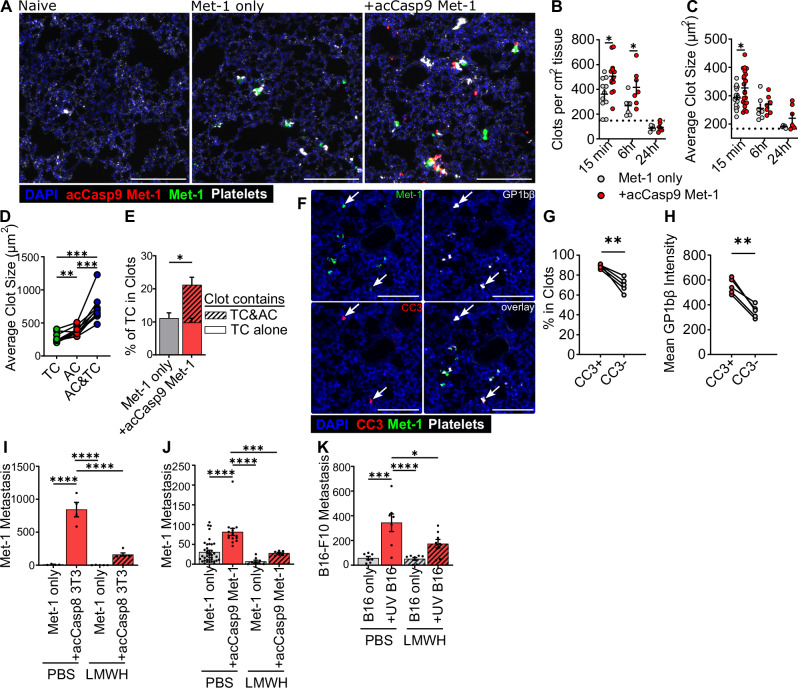
Apoptotic cells promote platelet aggregation on tumor cells, and the effect of apoptotic cells on metastasis depends on coagulation. Lungs were harvested at 15 min or indicated timepoints after I.V. injection and processed for fluorescent imaging (**A**–**H**). Met-1 tumor cells expressed ZsGreen (green), apoptotic acCasp9 Met-1 expressed mCherry (red), platelets are stained with αGPIbβ (white, **A**), cleaved caspase-3 (white, **F**) scale bar = 200 µm (**A**–**H**). Dotted line is the mean of naive lung controls (**B**, **C**). Average size of clots that contained either an Apoptotic cell (AC), Tumor cell (TC), or an AC and TC was quantified (**D**), and lines connect values from individual lung sections (**D**, **G**, **H**). Striped portion of the bar is the % of tumor cells in a clot that also contains an apoptotic cell (**E**). Low-molecular-weight heparin (LMWH) was administered s.c. at −4, 0, and 24 h in relation to I.V. tumor cell injection and metastasis quantified after 14 days (**I**–**K**). Error bars represent SEM, dots are multiple slices of lung from *n* = 3–4 biological replicates (**B**–**H**), or dots are biological replicates (**I**–**K**). Analysis with unpaired *T*-tests (**B**, **C**, **E**), paired *T*-test (**G**, **H**), or by ordinary one-way ANOVA with Tukey’s multiple comparisons test (**D**, **I**–**K**).

Platelets play an essential role in establishing the early metastatic niche and are critical for the survival of tumor cells in circulation^3–5,10^. Given that apoptotic cells promote metastasis by improving early CTC survival, we sought to address whether apoptotic cells influenced platelet aggregation on tumor cells. We compared platelet clots in the lung following injection of tumor cells alone or tumor cells mixed with apoptotic cells. We observed the rapid formation of platelet clots in the lungs after I.V. injection and noticed the clots were often associated with tumor cells and apoptotic cells (Fig. 3A). In mice that received tumor cells plus apoptotic cells we observed significantly more platelet clots as well as a larger average size of platelet clots at 15 min post-injection (Fig. 3B, C). Platelet clots formed rapidly after tumor cell injection and resolved by 24 h (Fig. 3B, C). Clots that formed around an apoptotic cell were larger than those around a tumor cell, while clots containing both a tumor cell and an apoptotic cell were the largest (Fig. 3D).

Considering that in our model system, the effect of apoptotic cells requires their spatial association with tumor cells in the vasculature, we hypothesized that coinjecting apoptotic cells may increase platelet aggregation on nearby tumor cells. Indeed, a higher percentage of tumor cells were located in platelet clots in mice coinjected with apoptotic cells than in mice receiving only tumor cells, and these clots also contained apoptotic cells (Fig. 3E). This indicates that apoptotic cells enhance platelet aggregation on tumor cells.

As mentioned previously, a portion of tumor cells themselves undergo apoptosis following arrest in the lung vasculature, so the difference in the quantity of clots in mice receiving tumor cells and apoptotic cells could be a result of the overall higher quantity of total cells seeding the lung. We therefore also measured platelet aggregation on tumor cells injected alone, comparing platelet aggregates around live or apoptotic tumor cells using the apoptotic marker, cleaved caspase-3 (CC3+) (Fig. 3F). We found that a higher percentage of CC3+ tumor cells were found within platelet aggregates compared with CC3- tumor cells (Fig. 3G). Additionally, the intensity of platelet staining on CC3+ tumor cells compared to CC3- tumor cells was significantly higher (Fig. 3H). This suggests that tumor cells undergoing apoptosis in the vasculature promote platelet aggregation.

We next sought to address whether coagulation was required for the prometastatic effects of apoptotic cells. To do this, we treated mice with the anti-coagulant Low-Molecular Weight Heparin (LMWH) prior to I.V. injection of tumor cells alone or tumor cells and apoptotic cells. In both Met-1 and B16-F10 metastasis models, we observed that treating mice with LMWH reduced the effect of apoptotic cells on metastasis (Fig. 3I–K). This suggests that coagulation is essential for apoptotic cells to promote metastasis.

### Apoptotic cells have enhanced procoagulant activity

**Fig. 4:**
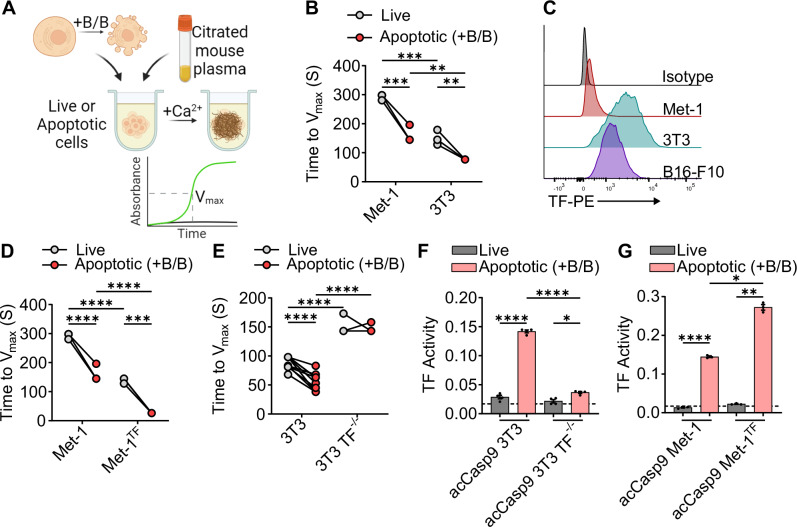
Apoptotic cells have enhanced procoagulant activity. Time to fibrin clot formation was measured as the maximum change in absorbance (Time to V_max_) after adding Ca^2+^ to citrated mouse plasma containing live or apoptotic cells; lower values indicate more rapid clot formation (**A**, **B**, **D**, **E**). Met-1 or 3T3 expressing acCasp9 were left untreated or treated with B/B for 2 h (**A**, **B**, **D**–**G**). TF-PE fluorescent intensity was measured by flow cytometry (**C**). TF activity was measured as the absorbance at 30 min after addition of live or apoptotic cells to mouse FVII, human FX, and chromogenic FXa substrate (**F**, **G**). Circles connected by lines are matched serum samples, and statistical analysis is two-way ANOVA with Tukey’s multiple comparison test (**B**, **D**, **E**). Dotted line represents the absorbance of a control well containing all assay components without cells added; statistical analysis is Welch’s ANOVA with Dunnett’s multiple comparisons test, and error bars represent SEM (**F**, **G**). Diagram created in BioRender. Oberst, A. (2025) https://BioRender.com/fa409ue (**A**).

Given our in vivo data indicating a role for coagulation in the promotion of metastasis by apoptotic cells, we sought to directly assess the procoagulant activity of apoptotic cells in vitro. To do this, we developed an assay based on the prothrombin time coagulation test, a test that measures how fast a clot forms within plasma^47^. We quantified how rapidly live or apoptotic cells formed a fibrin clot in citrated, platelet-poor plasma by measuring the change in absorbance after addition of Ca^2+^ (Fig. 4A). A lower time to clot formation indicates higher procoagulant activity. When Met-1 or 3T3 cells were stimulated to undergo apoptosis, the apoptotic cells were faster at inducing a fibrin clot compared to their live cell counterparts, as evidenced by a lower time to reach V_max_ (Fig. 4B). Notably, 3T3 cells were overall faster at initiating coagulation compared with Met-1 cells, mirroring the more robust promotion of metastasis observed upon coinjection of apoptotic 3T3 cells as compared to apoptotic Met-1 cells in vivo.

Plasma contact with the cell surface protein TF initiates the extrinsic coagulation cascade^48^. High TF expression on tumor cells has been linked with cancer-associated thrombosis and metastatic progression^11,49–51^. We wondered if the difference in coagulation rates of Met-1 vs. 3T3 cells could be due to differential expression of TF. Consistent with this possibility, Met-1 cells exhibited substantially lower expression of TF than 3T3 or B16-F10 cells (Fig. 4C). We therefore generated TF knockout 3T3 cells (3T3 TF^−/−^) or TF-overexpressing Met-1 cells (Met-1^TF^) to test whether TF was responsible for their procoagulant activity. Indeed, we observed that the rate of fibrin clot formation induced by each cell type correlated with TF expression. Overexpressing TF on Met-1 cells increased their rate of plasma clot formation (Fig. 4D) while ablating TF on 3T3 cells reduced their rate of plasma clot formation. Notably, ablation of TF on 3T3 cells abrogated the higher procoagulant activity of apoptotic cells compared to live cells (Fig. 4E). This indicates that the speed of clot formation by apoptotic cells depends on TF-mediated coagulation cascades.

TF activates coagulation by forming a complex with Factor VIIa, which catalyzes the conversion of Factor X to Factor Xa. However, TF often exists in an inactive state, termed encrypted TF, on resting cells, which prevents efficient catalysis of this reaction^52^. While the exact mechanisms controlling TF decryption remain incompletely defined, various cell perturbations, including those that induce phosphatidylserine (PS) externalization, can reveal full TF procoagulant activity^52^. We therefore directly examined TF activity on live vs. apoptotic cells by assaying the rate of Factor Xa generation in the presence of Factor VII and Factor X^53,54^. TF on live cells was largely unable to generate Factor Xa, irrespective of its expression level, suggesting that TF is in the encrypted state on live Met-1 and 3T3 cells. Inducing apoptosis significantly increased TF activity on these cells (Fig. 4F, G). The magnitude of TF activity after induction of apoptosis was dependent on the level of TF expression. Overexpressing TF on acCasp9 Met-1 or knocking it out on acCasp9 3T3 led to a corresponding increase or decrease of TF activity, respectively (Fig. 4F, G). Interestingly, necrotic and necroptotic cells also had robust procoagulant activity as measured by TF expression, activity, time to V_max_, and number of clots in vivo (Supplementary Fig. 8). Together, these in vitro data suggest that inducing apoptosis leads to TF decryption, resulting in enhanced procoagulant activity of apoptotic cells.

### Phosphatidylserine exposure promotes the coagulant activity of apoptotic cells

**Fig. 5:**
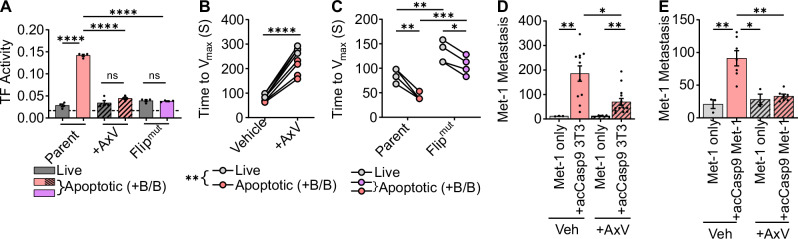
Phosphatidylserine exposure promotes the coagulant activity of apoptotic cells. acCasp9 3T3 were left untreated or treated with B/B for 2 h (**A**–**C**). Annexin V (AxV) was added to cells in a Ca^2+^ containing buffer and incubated on ice for 30 min (**A**, **B**, **D**, **E**). TF activity was measured as the absorbance at 30 min after addition of live or apoptotic cells to mouse FVII, human FX, and chromogenic FXa substrate (**A**). Time to fibrin clot formation was measured as the maximum change in absorbance (Time to V_max_) after adding Ca^2+^ to citrated mouse plasma containing live or apoptotic cells; lower values indicate more rapid clot formation (**B**, **C**). Surface lung metastasis was quantified at 14 days post I.V. injection (**D**, **E**). Dotted line represents the absorbance of a control well containing all assay components without cells added (**A**). Statistical analysis is Welch’s ANOVA with Dunnett’s multiple comparisons test; error bars represent SEM (**A**, **D**, **E**). Circles connected by lines are matched serum samples, and statistical analysis is two-way ANOVA with Tukey’s multiple comparison test (**B**, **C**).

PS externalization is an important mediator of thrombosis that occurs during platelet activation and endothelial damage and contributes to other pathological thrombotic events. PS externalization has been described as a mechanism for decrypting TF procoagulant activity as well as providing a negatively charged surface for assembly of the FXa-Va-prothrombinase coagulation enzyme complex^52,55–57^. PS externalization is also a canonical feature of the apoptotic plasma membrane; we therefore investigated whether the procoagulant activity of apoptotic cells was PS-dependent. We employed two distinct strategies to target PS. First, to physically block PS exposed on the outer membrane, we treated cells with saturating concentrations of the PS-binding protein Annexin V (AxV). We also generated apoptotic cells that do not externalize PS upon caspase activation by deleting the caspase-activated scramblase XKR8 and expressing a caspase-resistant version of the flippase ATP11c^58^ (acCasp9 3T3 Flip^mut^) (Supplementary Fig. 9). Blocking PS with AxV completely abrogated the ability of TF to generate Factor Xa in the presence of Factor VII and Factor X (Fig. 5A). acCasp9 3T3 Flip^mut^ cells, unable to externalize PS, also lacked TF activity after induction of apoptosis (Fig. 5A). These data suggest that TF decryption is dependent on PS externalization during apoptosis.

To examine whether the full procoagulant activity of apoptotic cells was dependent on PS externalization, we next tested these PS blocking strategies in the plasma clot assay. AxV treatment significantly reduced the plasma coagulation rate, but apoptotic cells treated with AxV still induced clotting faster than live cells treated with AxV (Fig. 5B). acCasp9 3T3 Flip^mut^ cells also retained an increased plasma coagulation rate upon caspase activation, despite having reduced overall procoagulant activity compared to parental cells (Fig. 5C). Together, this indicates that while TF decryption and maximal procoagulant activity on apoptotic cells are dependent on PS, apoptotic cells have residual, PS-independent procoagulant activity.

We next tested whether blocking PS resulted in reduced metastasis in vivo. We found that treatment of apoptotic cells with AxV significantly reduced their ability to promote metastasis (Fig. 5D, E). We hypothesized that increased CTC survival was a result of PS-dependent coagulation. The early benefit of apoptotic cells on tumor cell survival at 6 and 24 h was indeed reduced by treating cells with AxV (Supplementary Fig. 10A, B). Additionally, coinjecting necroptotic cells, which also expose PS on their outer membrane and have procoagulant activity^59^ (Supplementary Fig. 9), increased tumor cell survival at 24 h (Supplementary Fig. 10C). This suggests that coagulation initiated by dying cells benefits early CTC survival.

### TF expression is required for apoptotic cells to promote clotting in vivo and enhance metastasis

**Fig. 6:**
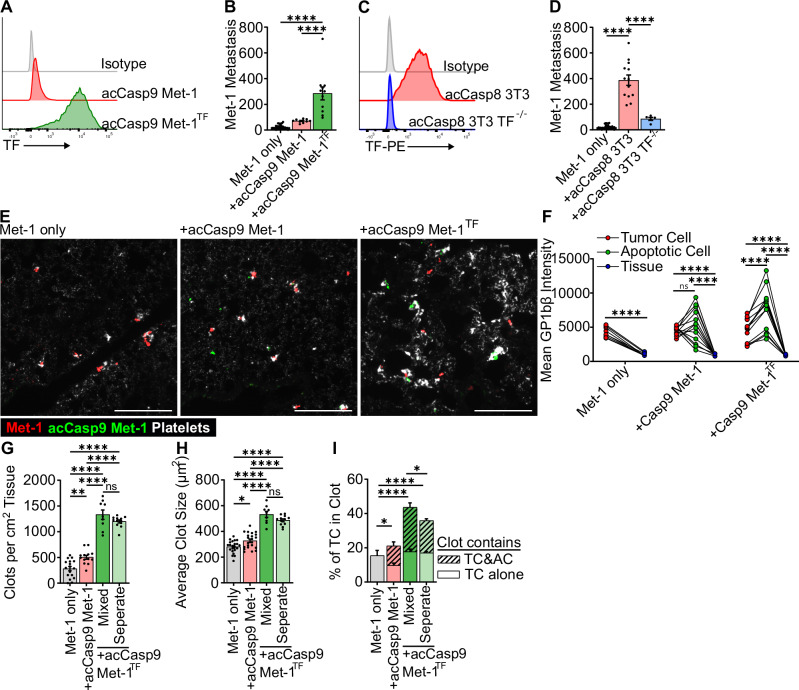
Tissue factor is responsible for the magnitude of apoptotic cell-enhanced metastasis and platelet clotting in the lung. TF-PE fluorescent intensity was measured by flow cytometry on live cells (**A**, **C**). Surface lung metastasis was quantified at 14 days post I.V. injection (**B**, **D**). Lungs were harvested for fluorescent microscopy 1 h after I.V. injection, Cells were mixed up to 1 h in advance and injected in a single bolus (mixed), or injected in two sequential injections into opposite tail veins (separate), acCasp9 Met-1 cells were stained with CFSE (green), Met-1 cells expressed mCherry (red), platelets are stained with αGP1bβ (white), scale bar = 200 µm (**E**–**I**) Lines connect values from individual lung sections (**F**). Striped portion of the bar is the % of tumor cells in a clot that also contains an apoptotic cell (**I**). Dots are biological replicates (**B**, **D**) or multiple slices of lung from *n* = 3–4 biological replicates (**F**–**I**). Statistical testing is Ordinary one-way ANOVA with Tukey’s multiple comparisons test (**B**, **D**, **F**–**H**) or Welch’s ANOVA with Dunnett’s multiple comparisons test (**I**). Error bars represent SEM.

Given the differential expression levels of TF on Met-1 vs. 3T3 cells (Fig. 4C) and the corresponding difference in magnitude of their effect on coagulation and metastasis (Figs. 1 and 4), we next sought to directly assess the role of TF in the promotion of metastasis by apoptotic cells. TF overexpression on apoptotic Met-1 cells increased their effect on metastasis to a level comparable to apoptotic 3T3 (Fig. 6A, B). Conversely, TF knockout reduced the effect of apoptotic 3T3s to a level comparable to that observed with apoptotic Met-1 (Fig. 6C, D). Consistent with apoptotic 4T1 cells minor effect on metastasis (Fig. 1H), TF expression on 4T1 cells was also very low, similar to that of Met-1 cells (Supplementary Fig. 11). Together, this suggests that TF expression and the procoagulant activity of apoptotic cells determine the magnitude of effect apoptotic cells have on promoting metastasis.

Given our previous observation that apoptotic cells promoted platelet aggregation at metastatic sites (Fig. 3), we next assessed the role of TF on apoptotic cells in this process. We found that the lungs of mice coinjected with TF-expressing apoptotic cells contained large platelet clots (Fig. 6E). The intensity of platelet staining associated with apoptotic Met-1 cells overexpressing TF was significantly higher than that observed on unmanipulated Met-1 tumor cells (Fig. 6F). Increasing TF expression on apoptotic cells significantly increased the number and size of clots found in the lung (Fig. 6G, H). We did not observe an increase in tumor cell quantity at 1 h after coinjection of TF-expressing apoptotic cells, suggesting that increased clotting in the presence of apoptotic cells does not result in increased arrest of tumor cells in the lungs. Rather, the formation of robust platelet clots by TF-expressing apoptotic cells correlates with increased survival of tumor cells at 24 h (Supplementary Fig. 12A–C). This suggests that expression of TF on apoptotic cells drives robust platelet activation during vascular dissemination and contributes to increased tumor cell survival.

Finally, we assessed the role of TF-overexpressing apoptotic cells in promoting aggregation of platelets directly on Met-1 tumor cells with low TF expression. We quantified the number of platelet clots containing solitary tumor cells or tumor cells co-localized with apoptotic cells. Compared to the small increase in tumor cells within a clot when coinjected with TF-low apoptotic cells, TF-high apoptotic cells led to a substantial increase in the percentage of unmanipulated Met-1 tumor cells found within clots. This increase was reflected in the portion of tumor cells localized to a clot that also contained a TF-expressing apoptotic cell, suggesting that TF expression on apoptotic cells can act in trans to promote platelet aggregation on tumor cells that have low TF expression (Fig. 6I).

We also examined whether injecting apoptotic cells separately from tumor cells, into opposite tail veins, had an effect on the localization of tumor cells and apoptotic cells within clots. While the overall size and number of clots were not affected by injecting apoptotic cells and tumor cells separately, the frequency of tumor cells located with an apoptotic cell and surrounded by platelets was decreased (Fig. 6G–I). This indicates that when apoptotic cells and tumor cells are injected separately, they are less likely to localize together within clots. Because injecting tumor cells and apoptotic cells in separate veins reduces their effect on metastasis (Fig. 2C, D) and the effects of apoptotic cells are dependent on their ability to trigger coagulation through TF (Figs. 4 and 6), we postulate that tumor cells localized together in a clot with an apoptotic cell may be more likely to survive and form a metastatic nodule. Together, these findings identify a previously unappreciated role for apoptotic cells in promoting metastasis, and suggest that TF expression by apoptotic tumor cells is a key contributor to this process.

## Discussion

Killing tumor cells by apoptosis is a central goal of most tumor therapies. Counterintuitively, however, an expanding body of evidence suggests that the presence of apoptotic cells, from either tumor or stromal origin, may correlate with increased tumor growth and negative clinical outcome. While apoptotic cell death within tumors is known to promote tumor cell proliferation, angiogenesis, and pro-tumor macrophage polarization^60–62^, a putative role of apoptotic cells throughout the metastatic cascade has not been well defined. Here, we report a striking role for apoptotic cells in facilitating metastasis by supporting early CTC survival. We find that the effect of apoptotic cells on metastasis depends on the procoagulant activity of TF, which is induced upon PS exposure on apoptotic membranes.

While our reliance upon the I.V. metastasis model allowed us to observe the striking effect of apoptotic cells in circulation, it does not provide insight into the effects of apoptotic cells at other stages of the metastatic cascade and does not closely mimic some physiological features in human patients. In patients, CTC clusters are very rare and shed slowly over time. The I.V. model is instead a single bolus of tumor cells seeding the lung simultaneously. This allowed us to rapidly and precisely quantify the effects of apoptotic cells on CTC survival, as these events would be expected to be much more infrequent and therefore difficult to study in the context of spontaneous metastasis from either human or murine tumors. Advancements in techniques to observe rare CTCs will be useful for future research to confirm that the effects of apoptotic CTCs observed herein persist when tumor cells are spontaneously shed from a primary tumor.

In imaging the early seeding of CTCs in the lungs, we observed that apoptotic cells promote platelet aggregation, resulting in increased clotting on tumor cells. We hypothesize that the observed colocalization of apoptotic cells and tumor cells within clots may be responsible for supporting CTC survival. Platelet aggregation on tumor cells is important for shielding tumor cells from NK cell cytotoxicity and reducing shear stress, two significant contributors to early CTC demise^3–8^; our findings suggest that the presence of apoptotic cells offers a mechanism for CTCs to activate coagulation to survive these pressures. Extrinsic coagulation mediated by TF is rapid, and intravascular aggregation of CTCs is rare^14^; thus, when apoptotic and tumor cells do not enter circulation together—modeled herein as separate injections into opposing tail veins—apoptotic cell-induced coagulation would not occur in proximity to a tumor cell, thus abrogating the metastatic benefit conferred by apoptotic cells. CTC clusters have been shown to metastasize significantly more effectively than individual CTCs. CTC clusters disseminate collectively, entering circulation as a single unit, and have been shown to contain apoptotic cells^14,15,37^. Thus, CTC clusters represent a physiological context in which an apoptotic cell could be localized with a tumor cell in circulation. While various prometastatic characteristics of CTC clusters have been defined, such as stemness, apoptosis resistance, and immune escape^13,16^, the effects of apoptotic cells within CTC clusters have not been investigated. An intriguing possibility for one metastatic advantage conferred by CTC clusters is that a portion of cells in clusters could undergo apoptosis, thus supporting the survival of other cells within the cluster. Technological advances in detecting and analyzing CTC clusters could shed light on the clinical and physiological relevance of apoptotic cells as a component of CTC clusters.

The ability of tumor cells to induce extrinsic coagulation through TF expression or platelet aggregation through platelet activating ligands has been extensively described^3^. We find that apoptotic cells initiate robust extrinsic coagulation through PS-mediated TF decryption. By measuring procoagulant activity in platelet-poor plasma, we find that TF expression is necessary to induce fibrin clot formation in the absence of platelets. It appears that TF-mediated coagulation drives subsequent platelet aggregation in vivo, since we see a reduction in platelet aggregation when using TF^−/−^ cells. An important role of TF expression on CTCs that successfully metastasize has been described^50,63^; however, our work suggests that TF-mediated coagulation can also act in trans to support CTC survival. Thus, tumor cells with low TF expression, such as Met-1 cells, may depend on apoptosis of other cells as a source of TF activity to facilitate coagulation during vascular dissemination. Fibroblasts have been observed in association with CTCs^37,64,65^ and are a potential source of TF-mediated coagulation during metastasis that may be particularly relevant for the survival of TF-low tumor cells in circulation.

Our findings bolster the concept that apoptotic cells are an important procoagulant mechanism in various health and disease settings^66^. TF activity regulated through phosphatidylserine exposure has been described in other disease contexts such as endotoxemia^53,67,68^ and vessel injury^54,69^. Most relevant to our model is the consideration of apoptotic cells as a contributing factor to cancer-associated thrombosis^70–73^. Interestingly, the risk of venous thromboembolism increases following chemotherapy^74–76^, a regimen that causes massive apoptosis throughout the body. While chemotherapy has numerous effects beyond induction of apoptosis, our data clearly demonstrate that direct activation of caspases in both tumor and non-tumor cells is a source of procoagulant activity, suggesting that apoptotic cells may be an important contributor to hypercoagulation following chemotherapy.

While our work establishes apoptotic cells as a source of TF and PS-induced coagulation that supports CTC survival in the vasculature, other sources of TF and PS exist, and the differential effects of these unique activators of coagulation throughout the metastatic cascade remain an open question. Extracellular vesicles are a source of procoagulant activity and can exhibit prometastatic properties^73,77,78^. Apoptosis results in membrane blebbing and release of extracellular apoptotic vesicles, but whether there are distinct outcomes depending on whether EVs are derived from live or apoptotic cells is not clear. Tumor cells themselves have elevated surface PS, and targeting PS as a therapeutic strategy has been promising^30,79–84^. We observed PS externalization on tumor cells in circulation, and interpreted this observation as an early marker of apoptosis. However, it is also possible that deformation and shear stress in circulation result in Ca^2+^ flux, which could activate the TMEM family of PS scramblases^59^, resulting in PS externalization on live cells. Technical limitations in visualizing externalized PS in vivo prevent observation of PS externalization in combination with platelet aggregation, so we cannot yet delineate the potential contributions of PS exposure on live cells to coagulation in vivo.

Necrotic cells also have procoagulant activity^67,85–87^, and we observed that they enhance tumor cell survival after initial lung seeding, yet these cells do not promote metastasis. One possibility is that necrotic cells shift the activation of metastasis-associated myeloid cells towards an anti-metastatic phenotype, thereby reducing the success of surviving tumor cells. Alternatively, we could interpret these data as indicating that coagulation induced by dying cells is necessary but not sufficient for dying cells to promote metastasis; apoptotic cells may influence the metastatic process beyond their role in inducing coagulation. We note, however, that our data and published findings suggest apoptosis as a predominant mechanism of CTC death during metastasis; while assessment of effects of necrotic or necroptotic death in this context informs our understanding of the mechanisms of metastasis promotion, it is unlikely to reflect physiological conditions.

While our data highlight the key role of apoptotic cells in initiating coagulation to support metastasis, we are intrigued by the possibility that additional mechanisms exist by which apoptotic cells influence the metastatic process. Apoptotic cells alter their surrounding microenvironment in profound ways. Molecules such as fractalkine, lactotransferrin, and prostaglandin E_2_ are released by apoptotic cells to recruit macrophages and can also act on tumor cells to promote survival and proliferation^61,88–91^. Upon caspase activation, apoptotic cells release various anti-inflammatory metabolites from pannexin-1 channels^92^. Interestingly, ATP released through pannexin-1 was demonstrated to have pro-survival signaling in an autocrine manner on CTCs^93^. In the context of a CTC cluster, these various signaling molecules released from apoptotic cells could act in a paracrine fashion to support nearby tumor cells. Furthermore, the interactions between apoptotic cells and macrophages have been extensively characterized as leading to tissue-repair macrophage phenotypes^29^. Previous work has demonstrated that efferocytosis of apoptotic cells within tumors can polarize macrophages, which produce TGF-β and other wound-healing cytokines to facilitate metastatic dissemination^27^. CTCs release microparticles upon early arrival in the lung vasculature that are sampled by myeloid cells and guide their differentiation^40^. We observe rapid uptake of apoptotic cells by phagocytes in the lungs, and in the Met-1 model, macrophages do have an important role in facilitating metastasis. However, whether the interaction between apoptotic cells and macrophages contributes to the differentiation of metastasis-associated macrophages is an open question. The downstream effects of apoptotic cells beyond their most proximal role in inducing coagulation during hematogenous dissemination are an interesting area for future investigation.

Decades of work have been aimed at finding mechanisms to kill tumor cells, primarily through inducing apoptosis. Our work supports a growing body of evidence that apoptosis in the context of cancer can have adverse pro-tumor effects. Eliminating tumor cells, primarily through apoptotic cell death, will remain the primary goal of cancer therapeutics. However, understanding the subsequent effects of apoptosis on cancer progression could inform additional interventions countering the pro-tumor secondary effects of apoptosis. For example, blocking apoptosis by inhibiting caspase activation results in the conversion of apoptosis to an inflammatory form of cell death capable of enhancing anti-tumor immunity^94^. Whether this strategy could also reduce metastasis should be addressed. Because we define apoptotic cells as an important source of coagulation and show that LMWH treatment reduces the effect of apoptotic cells in promoting metastasis, targeted anti-coagulants are another possible therapeutic strategy. Temporal administration of anti-coagulants or the use of PS or TF blocking agents during times where high levels of apoptosis are expected, such as during cytotoxic therapy, could specifically target coagulation initiated by apoptotic cells. While the use of anti-coagulants has been proposed as a potential therapeutic to reduce metastatic spread^95,96^, they are not yet broadly used as anti-metastatic agents. The safety and efficacy of anti-coagulants in patients at risk for cancer-associated thrombosis is being evaluated^97,98^; our data suggest that an interesting additional parameter to monitor in these trials could be progression to metastatic disease.

Building upon clinical and experimental evidence that apoptosis can have unintended pro-tumor consequences, this work demonstrates a direct mechanism by which the presence of apoptotic cells during vascular CTC dissemination promotes metastatic disease. By activating coagulation through PS-dependent TF decryption, apoptotic cells support the survival of tumor cells in circulation. These findings emphasize that it is important to consider not only the cells that directly form metastatic tumors, but also the effects of cells that die throughout the metastatic cascade, opening up new avenues for investigation into the process of metastasis and possibilities for therapeutic intervention.

## Methods

### Sex as a biological variable

Our study examined only female animals. It is unknown whether the findings are relevant for male mice.

### Cell culture

Cell lines were cultured at 37 °C with 5% CO2 in Dulbecco’s modification of Eagle medium (DMEM) supplemented with 10% (v/v) fetal bovine serum (FBS), 2 mM l-glutamine, 10 mM Hepes, and 1 mM sodium pyruvate (complete DMEM). Cell lines were passaged every 1–3 days and used for experiments within 2 weeks after thawing from liquid nitrogen stocks. Cells were regularly tested and remained negative for mycoplasma contamination. Adherent cells were detached from the plate using Trypsin-EDTA (0.25%). MEFs were derived from day 15.5 embryos of B6/J or FVB/N pregnant mice as previously described^99^. MEFs were immortalized by transforming with a lentivirus containing SV40 Large T antigen (iMEF). B16-F10 (CRL-6475) and NIH/3T3 (CRL-1658) cells were purchased from ATCC. Met-1 cells were derived from mammary carcinomas in FVB/N-Tg(MMTV-PyVmT) and kindly provided by Dr. Alexander Borowsky^31^. Met-1^TF^ cells were generated by lentiviral transformation with pCMV3-C-GFPSpark containing the mouse TF ORF (SinoBiological). NIH/3T3 TF^−/−^ cells were generated by lentiviral transformation with pRRL.U6.gRNA.MND.Cas9.2A.Hygro containing the guide RNA sequence CTCGTCTGTGAGGTCGCACT. Flip^mut^ cells were generated by lentiviral transformation with pRRL.U6.gRNA.MND.Cas9.2A.Hygro containing the guide RNA sequence targeting *XKR8*, CGTACTGGACAACGGCCCAC, and pMX.puro containing a mutant of ATP11c with D to A mutations in the three caspase cleavage sites, kindly provided by Dr. Shigekazu Nagata^58^.

### Mice

Female C57BL6/J (B6/J) mice and FVB/NJ mice (FVB) were purchased from the Jackson Laboratory and allowed to acclimate for at least 1 week before experiment initiation. Experiments were performed on 6–10-week-old mice. A *Rag2*^*−/−*^ breeding pair was purchased from the Jackson Laboratory and bred in-house for experiments. Cx3cr1-Cre^+/+^ and BELMO^Tg/WT^ mice were kindly provided by Dr. Kodi Ravichandran^41^. Cx3cr1-Cre^+/+^ dams were crossed with BELMO^Tg/WT^ heterozygous sires to generate littermate controls, which all contained Cx3cr1-Cre^+/+^ and a mixture of mice that contain one or no copies of the BELMO floxed transgene. Mice were housed under specific pathogen–free conditions at the University of Washington. All animals were maintained and used according to protocols approved by the University of Washington Institutional Animal Care and Use Committee (IACUC), under protocol number 4298-01 (PI: AO). We have complied with all relevant ethical regulations for animal use.

### Cell death induction

The activatable cell death systems have been described extensively previously^32–35^. Briefly, NIH/3T3 cell lines were transduced with lentiviral plasmid (pRRL) encoding activatable (“ac”) Caspase-8, Caspase-9, or RIPK3. pSLIK lentiviral vector was used for thyroid response element-controlled expression of acCasp9 in B16-F10 and Met-1 cell lines and GSDME in NIH/3T3 cells. pSLIK gene expression was induced by culturing cells in doxycycline (1 µg/ml; Sigma-Aldrich) for 18 h before harvesting for cell death induction. Programmed cell death was induced by incubating cells in complete DMEM with 1 mM B/B homodimerizer (Clontech) for 15 min at 37 °C. Cells were then washed with cold phosphate-buffered saline (PBS) before being resuspended in PBS at designated concentrations. Cells killed by freeze/thaw lysis were cycled between liquid nitrogen and a 37 °C water bath 3 times. Cells killed by UV irradiation were exposed to 100 mj/cm^2^ UV light using a UVP CL-1000 Ultraviolet Crosslinker. % Cell death was monitored using Sytox Green dye (Invitrogen) and Incucyte live cell imaging (Sartorius). Phosphatidylserine exposure was analyzed on a flow cytometer after staining cells with Fluorescent conjugated Annexin V (Invitrogen) and Propidium Iodide (Ebioscience) in Annexin Binding Buffer.

### Intravenous metastasis

1.5 × 10^5^ Met-1 tumor cells, 7 × 10^4^ B16-F10 cells, or 2 × 10^4^ 4T1 cells were suspended in PBS with an equivalent number of apoptotic cells unless otherwise indicated in the figure legend. One hundred microliters of this mixture was injected intravenously into the tail vein of mice after warming under a heat lamp for 5 min. B16-F10 metastasis was quantified 14 days after by counting black tumor nodules on the surface of all lung lobes. Met-1 and 4T1 metastasis was quantified by staining lung tissue with 15% India Ink intratracheally, then incubating lungs in Fekete’s solution for 24 h at 4 °C before counting white tumor nodules on the surface of all lung lobes. In all models, metastatic nodules of any size that were clearly visible on the lung surface under a 2× dissection microscope were counted as one nodule. The investigator counting lung metastasis was blinded to the experimental groups. For separate tail vein experiments, apoptotic cells and tumor cells were suspended in PBS, and 100 µL of each cell type was injected into the left and right tail veins, respectively. For experiments with tumor-bearing mice, 5 × 10^4^ B16.F10 cells were suspended in a 1:1 mixture of PBS and Matrigel Matrix High Concentration (Corning) and injected subcutaneously into the right flank of B6/J mice. Tumor volume was measured using digital calipers throughout the experiment (elliptical volume = width^2^ × height × 0.523)^100^ and mice were euthanized if tumors reached the maximum volume of 2000 mm^3^ as permitted by our IACUC protocol. Maximal tumor volumes were not exceeded in any of the animal experiments. 1 × 10^5^ B16.F10 cells were injected intravenously alone or with 1 × 10^5^ UV-irradiated B16.F10 cells on days 4, 5, and 6 post-tumor challenge. Metastasis was quantified at 14 days post-tumor challenge.

### Annexin V blocking of PS

After cells reached maximum PS exposure, 15 min for acCasp9 3T3 and 2 h for acCasp9 Met-1 (Fig. 1B), 25 µg/mL purified Annexin V (Biolegend) was added to cells in HBSS+Ca^2+^+Mg^2+^ and incubated on ice for 30 min prior to injection or use in coagulation or TF activity assays.

### Quantification of injected cell genomic DNA in the lung

The left lobe was dissected, rinsed in PBS, and placed in 1 mL DNAzol (Invitrogen). The tissue was homogenized using Precellys Tissue Homogenizer with ceramic beads. Genomic DNA was isolated from the homogenate according to the DNAzol reagent manual. The concentration of genomic DNA isolated was quantified using a spectrophotometer, and concentrations were normalized by diluting samples in nuclease-free water. Quantitative Taqman probe-based PCR was used to quantify the PyMT transgene or other ectopically expressed transgenes (ZsGreen, GFP, mCherry). Primer/Probe sequences can be found in Supplementary Table 1. The relative abundance of target genes was calculated as 2^−ΔΔCT^, where ΔCT = CT^Target^−CT^ptger2^ and ΔΔCT = ΔCT^sample^−Average ΔCT^initial^.

### Heparin treatment

Two hundred micrograms of LMWH, Enoxaparin sodium (Sigma-Aldrich) dissolved in 200 µL PBS was injected subcutaneously into the flank of mice at hours −4, 0, and 24 relative to I.V. tumor cell injection.

### NK cell depletion

Mice were administered 200 µg anti-NK1.1 clone PK136 (Bio X Cell) or isotype mouse IgG2a clone C1.18.4 (Bio X Cell) in 200 µL PBS intraperitoneally at 72 and 24 h prior to I.V. metastasis challenge.

### Flow cytometry

Lungs were rinsed in PBS, roughly dissociated with scissors, then incubated with 50 µg/mL Liberase TM (Sigma), 250 µg/mL DNAse I (Sigma) in HBSS with Ca^2+^ and Mg^2+^ for 35 min at 37 °C with gentle agitation. Tissue was homogenized with GentleMACS lung dissociation (Miltenyi Biotec) and strained through 70 µm cell strainers. Single cell suspensions were stained with fluorochrome-conjugated antibodies in PBS with 3% FBS +0.05% NaAzide. Antibodies used are CD45 clone 30-F11 (Biolegend), TF polyclonal (R&D Systems), CD3 clone 145-2C11 (BD Bioscience), NKp46 clone PK136 (Biolegend), MHCII clone M5/114.15.2 (eBioscience), Ly6G clone 1A8 (BD Biosciences), SiglecF clone E50-2440 (BD Pharmingen), CD19 clone 1D3 (BD Biosciences), CD90.1 clone OX-7 (BD Biosciences), NK1.1 clone PK136 (BD Biosciences), CD11c clone N418 (Biolegend), CD24 clone M1/69 (BD Biosciences), CD11b clone M1/70 (BioLegend), Ly6C clone AL-21 (BD Biosciences). Concentrations and catalog numbers for antibodies can be found in Supplementary Table 2. Zombie NIR Live/Dead stain (BioLegend) was used in myeloid characterization panels. Gating Strategy for Myeloid cells can be found in Supplementary Fig. 13. For PS detection, fluorescent conjugated Annexin V (Invitrogen) and Propidium Iodide (Ebioscience) were added to cells suspended in Annexin Binding Buffer prior to running on either a CantoRUO or Symphony flow cytometer (BD Biosciences). Data was analyzed with FlowJo software (TreeStar).

### Immunofluorescent imaging

Mouse lung tissue was fixed in a 1:3 dilution of Cytofix fixation buffer (BD) in PBS for 18 h, washed with PBS twice, then embedded in OCT medium and frozen. In some experiments, cells were stained with CFSE (Invitrogen) prior to injection. 20 µm slices were prepared with a Cyrostat on glass microscope slides and stained with primary and secondary antibodies in 1%BSA in PBT (PBS with 0.1% Triton X-1000) after blocking with 10% BSA in PBT. Antibodies used were anti-GPIbβ conjugated to Dylight 649 (X649, Emfret analytics); Rabbit anti-Cleaved Caspase-3 (Asp175, Cell Signaling Technology), and rabbit anti-mCherry polyclonal (Rockland). Slides were mounted with Aqua-mount (Epredia), and images were acquired on a Leica DMI6000 inverted microscope with a 10X LWD dry objective and a Leica DFC365 FX CCD camera. LASX software was used for image acquisition and mosaic tiling.

### Image analysis

ImageJ was used to quantify platelet clots, tumor cell numbers, and apoptotic cell numbers. Positive staining was defined using manual thresholding. Clots, tumor cells, and apoptotic cells were defined as stain-positive objects >200 µm^2^. QuPath was used to quantify spatial parameters such as colocalization of cells within clots and distances between cells using manual thresholding to define objects.

### Plasma coagulation assay

Blood was drawn from the left ventricle into syringes containing citrate-dextrose solution (Sigma-Aldrich) at a dilution of 9:1. Platelet-poor plasma was isolated after two sequential centrifugations at 5000 × *g* for 10 min. Plasma was stored at −80 °C and thawed only once before use in the assay. All reagents were brought to 37 °C for assay. 50 µL citrated platelet-poor plasma was aliquoted into wells of a 96-well polystyrene plate. 100,000 cells were added to wells in 50 µL PBS. Fifty microliters of 25 mM CaCl_2_ was added to allow the coagulation reaction to occur. Absorbance at 405 nm was measured every 15 s by the Syngery HT microplate reader at 37 °C with a 2-s shaking step in between each read. The time to the maximum change in absorbance was calculated (Time to V_max_).

### Tissue factor activity assay

Live cells or cells stimulated to undergo apoptosis (20,000 per well) were added to a 96-well plate. Hepes-buffered saline with BSA (1 mg/mL) containing 0.6 nM Mouse Factor VII (Biotechne), 125 nM Human Factor X (Prolytix), and 150 µM Factor Xa chromogenic substrate (Sigma-Aldrich) was added to each well. Absorbance at 405 nm was read every 3 min on a Biotek Synergy HTX microplate reader set to 37 °C with a shaking step before each reading. Absorbance readings were blanked to the absorbance of the well at 0 min.

### Statistics and reproducibility

GraphPad Prism was used to calculate statistical significance. Statistical tests are indicated in the figure legends. *,**,***,**** corresponds to 0.01 > *P* > 0.05, 0.001 > *P* > 0.01, 0.0001 > *P* > 0.001, *P* < 0.0001, respectively. Statistical tests and sample sizes are described in the figure legends. For data where less than two biological replicates were performed, statistics are not reported. Experimental means and standard deviation from preliminary experiments were used in a power calculation, with power set to 0.8 and type I error rate set to 5% to determine the required sample size. Mice were randomly assigned to experimental groups, and the investigator was blinded to the groups during data collection. No data were excluded from analysis, and all key experiments were repeated two or more times in independent experiments.

### Reporting summary

Further information on research design is available in the Nature Portfolio Reporting Summary linked to this article.

## Supplementary information

Transparent Peer Review file

Supplementary Information

Reporting Summary

## Data Availability

Values for all figures can be found on figshare 10.6084/m9.figshare.25872388.v2^101^.

## Materials availability

Reagents described in this manuscript are available from A.O. for research use under a materials transfer agreement from the University of Washington.
