# Supplementary material for: Apoptotic cells promote circulating tumor cell survival and metastasis
Source: Commun Biol. 2025 Jul 29;8:1121. doi: 10.1038/s42003-025-08541-7 (PMC12307979; doi:10.1038/s42003-025-08541-7)
Supplement: Supplementary file 2 — Supplementary Information [file 42003_2025_8541_MOESM2_ESM.pdf]

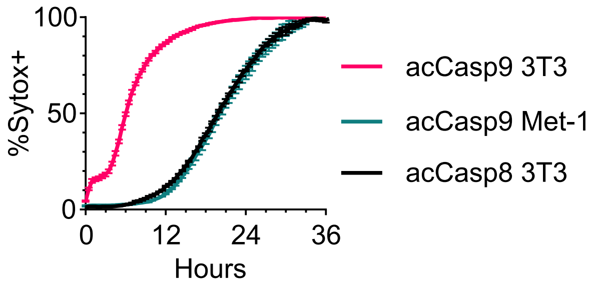

Supplementary Figure 1. Cell Death Kinetics of activatable apoptosis systems. acCasp9 3T3, acCasp9 Met-1 and acCasp8 3T3 were incubated with B/B and Sytox green dye. % Sytox+ cells were measured with Incucyte live cell imaging to measure membrane permeability.

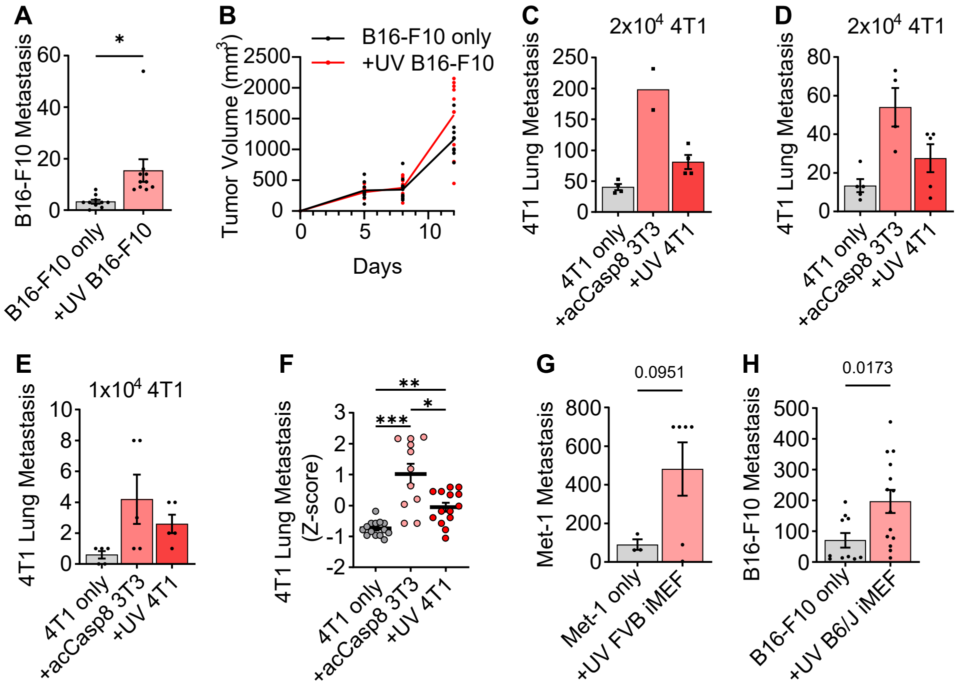

Supplementary Figure 2. Additional I.V. metastasis assays. B16-F10 tumor bearing mice were challenged with I.V. metastasis at days 4, 5, and 6 post tumor implantation. Tumors were measured daily and lung metastasis was quantified at 14 days post tumor implantation (A-B). The indicated quantity of 4T1 tumor cells were injected I.V. into Balb/C mice and surface lung metastasis quantified 14 days later. Three replicate experiments were performed (C-E) and Z-scores were calculated for statistical testing due to inter-experiment variability (F). MEFs were derived from FVB/NJ (G) or B6/J (H) mice and immortalized with SV40LT transduction (iMEF). iMEFs were UV irradiated and injected into mice at a 1:1 ratio with Met-1 (G) or B16-F10 (H). Surface lung nodules were quantified 14 days after I.V. injection. Lungs containing >700 surface metastasis were unable to be accurately quantified due to significant overlap of nodules, thus the maximum value of 700 was recorded in these cases. Dots are biological replicates, statistical testing is unpaired t-test (A, G-H) or Welch's ANOVA with Dunnett's multiple comparisons test (F).

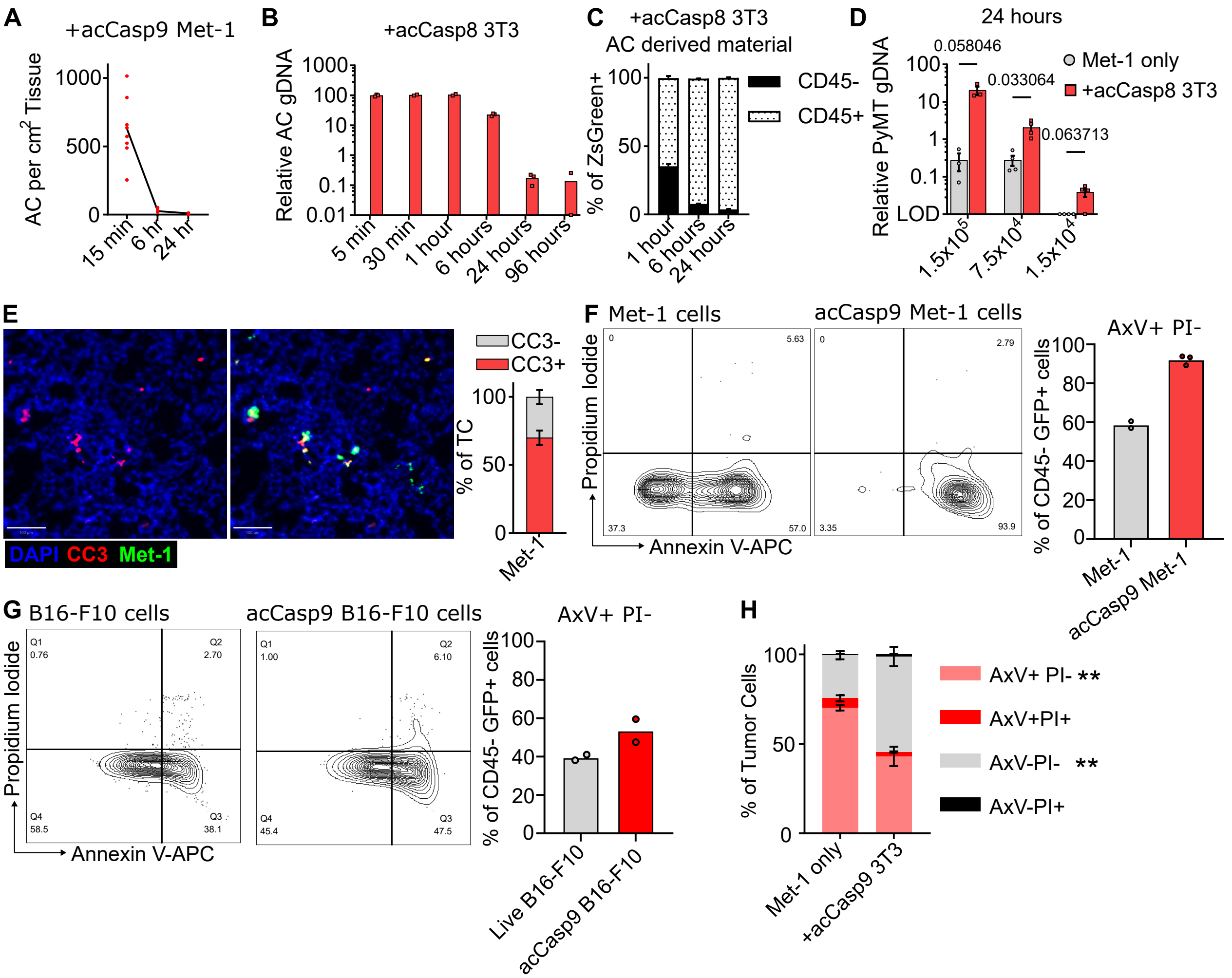

Supplementary Figure 3. Quantification of Apoptotic cell and Tumor cell persistence and state in the lung. Lungs were harvested at 1 hour or indicated timepoints for analysis by fluorescent microscopy (A,E), qPCR (B,D), or Flow Cytometry (C,F-H). Tumor cells expressed ZsGreen (A,E-H) and apoptotic cells expressed mCherry (A) or ZsGreen (B,C,F,G). Dots are biological replicates, statistical testing is unpaired t-test. AC=Apoptotic Cell, TC=Tumor Cell

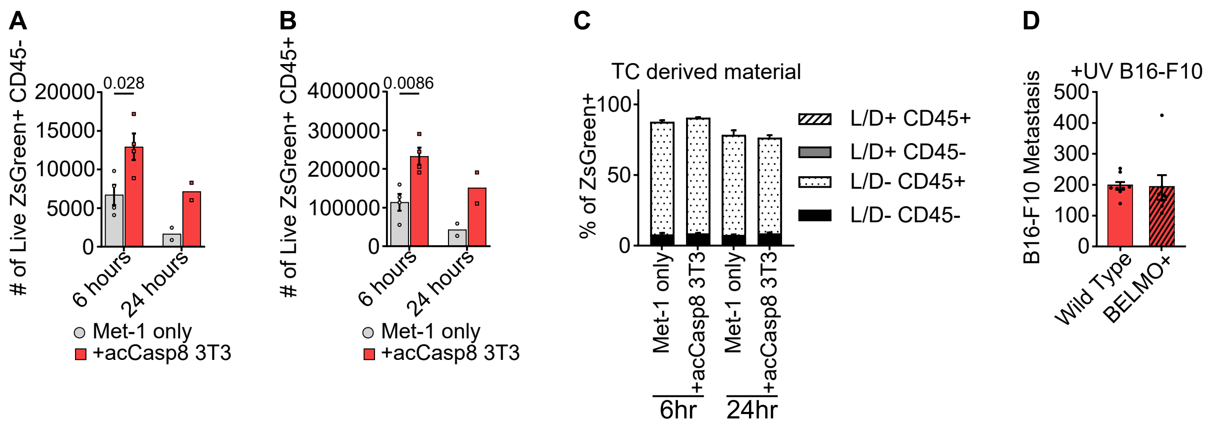

Supplementary Figure 4. Apoptotic cells do not limit phagocytic uptake of tumor cells. Lungs were harvested at indicated timepoints for Flow Cytometry analysis of phagocytic uptake of ZsGreen expressing Met-1 tumor cells. L/D=Zombie Live/Dead stain (A-C). B16-F10 metastasis was quantified 14 days after I.V. challenge of B16-F10 cells injected with UV irradiated B16-F10 cells in Cx3cr1-Cre$^{+/+}$ (Wild Type) or Cx3cr1-Cre$^{+/+}$ BELMO$^{Tg/WT}$ (BELMO+) mice (D). Dots are biological replicates, statistical testing is unpaired t-test.

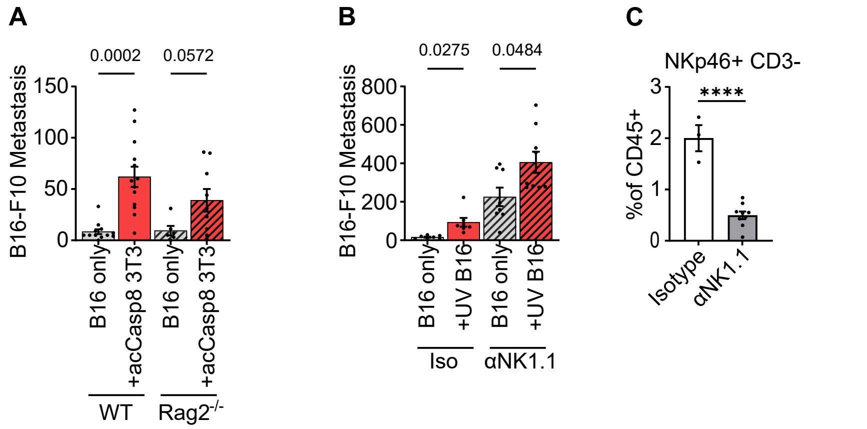

Supplementary Figure 5. Effects of apoptotic cells on B16-F10 metastasis in Rag2$^{-/-}$ and NK cell depleted animals. Apoptotic cells were injected at a 1:1 ratio with B16-F10 tumor cells. Surface lung nodules were quantified 14 days after I.V. injection (A-B). NK cell frequency of total CD45+ cells in the blood was measured 24 hours after I.V. injection of tumor cells to confirm NK cell depletion (C). Dots are biological replicates analyzed by Welch's ANOVA with Dunnett's multiple comparisons test (A-B) or unpaired t-test (C).

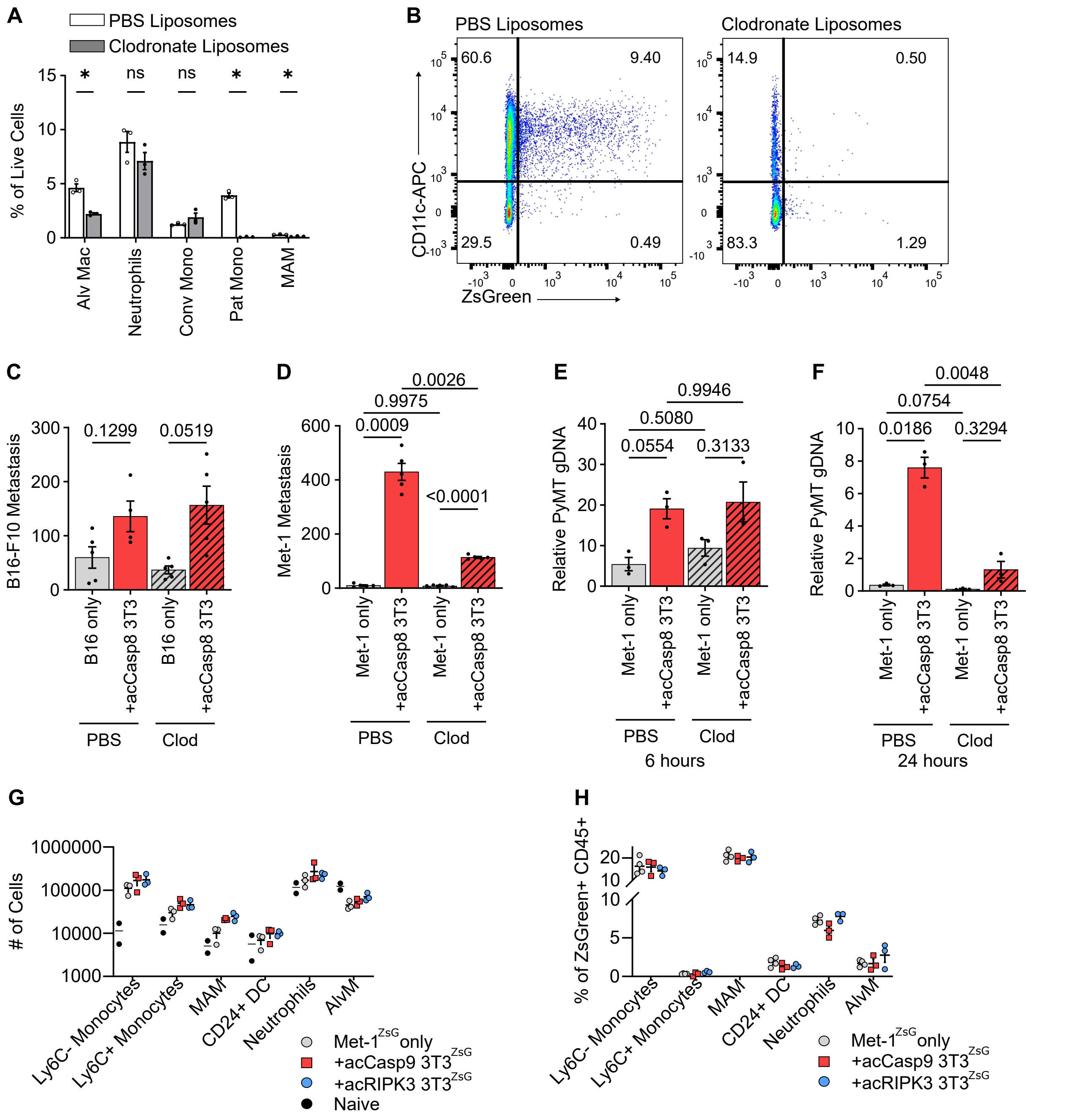

Supplementary Figure 6. Phagocyte depletion reduces metastasis in Met-1 model but not B16-F10. Depletion of phagocytic populations was quantified by Flow Cytometry 24 hours after I.V. injection of Met-1 cells and ZsGreen expressing acCasp8 3T3 (A-B). Surface metastasis was quantified 14 days after I.V. (C-D). Lung gDNA was processed at indicated timepoints and tumor cell quantity determined by qPCR (E-F). Myeloid cell populations were quantified 24 hours after I.V. using flow cytometry (G-H). Met-1, acCasp9 3T3, or acRIPK3 3T3 expressed ZsGreen (ZsG) to measure phagocytosis of tumor or dying cell material (H). Error bars represent SEM. Dots are biological replicates analyzed by unpaired t-test (A) or Welch's ANOVA with Dunnett's multiple comparisons test (C-F).

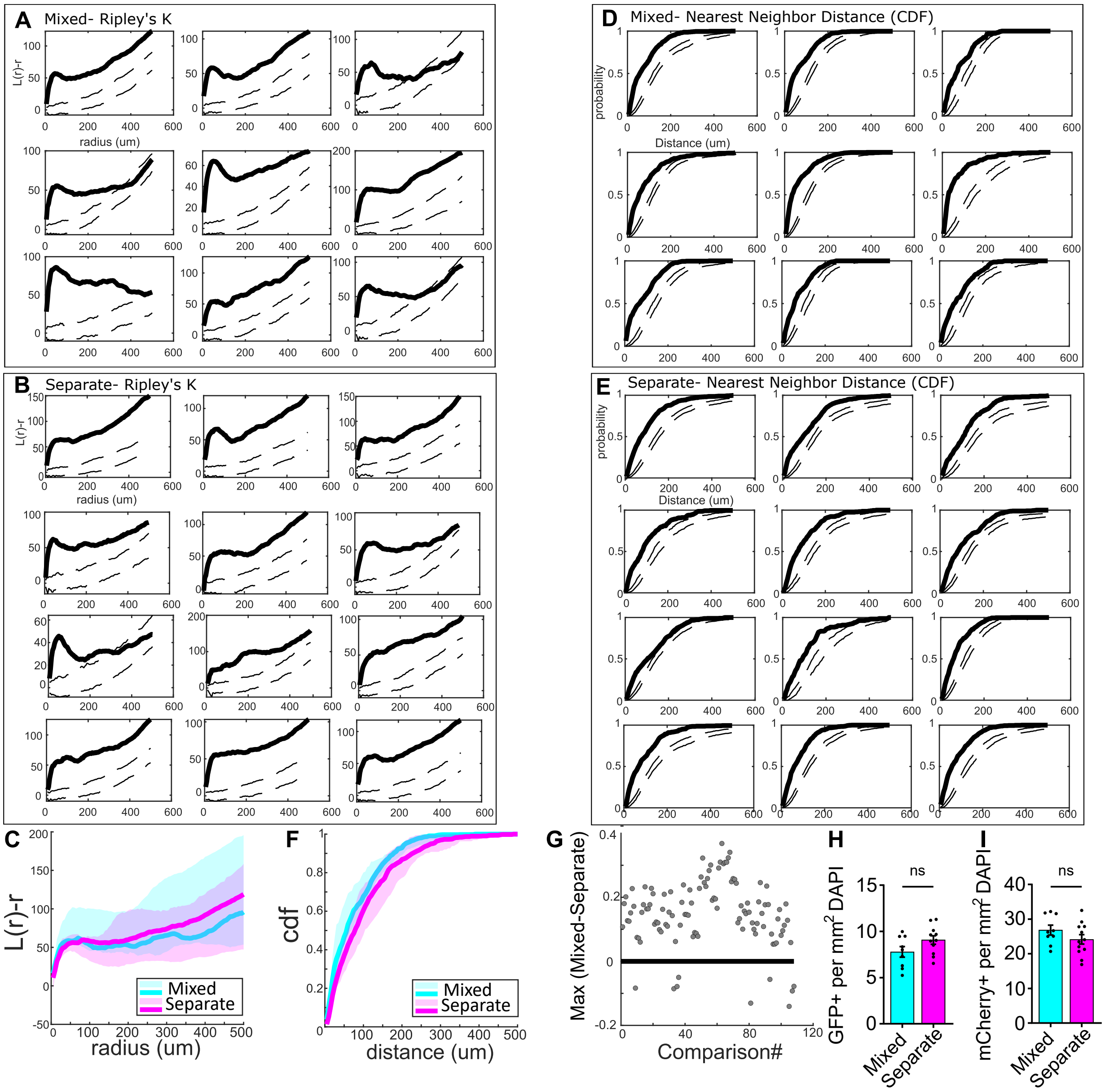

Supplementary Figure 7. Spatial analysis of tumor/apoptotic cell localization. Met-1 cells (mCherry+) and acCasp9 Met-1 (GFP+) were mixed up to one hour in advance (mixed) or injected in two sequential injections into opposite tail veins (separate). Lungs were harvested 1 hour after I.V. injection for fluorescent imaging. Bivariate Ripley K value was calculated for each experimental samples (solid black line) compared to random distributions (100 simulations, dashed black lines corresponding to 95% confidence intervals) (A-B) Ripley values (solid color) with 95% confidence interval (opaque) for each group was used to calculate two-sided p-values, P values range from 0.01 to 0.09 for radii between 5 and 65um (C). Cumulative distribution function (CDF) curves for each experimental sample (solid black line) reveal a positive association between apoptotic cells and tumor cells compared to random CDF (100 simulations, dashed black lines corresponding to 95% confidence intervals) (D-E). Median Mixed CDF is shifted to the left of median Separate CDF (p = 4.8e-5, Kolmogorov-Smirnov test), suggesting that cells clustering is more pronounced in Mixed samples (F). The max difference in CDF between every combination of samples from each experimental group was calculated. Positive differences indicate stronger cell clustering in Mixed group with mean ~ 0.15 [0.13 0.17] with p=1.5e-28 that the mean is not zero indicating that apoptotic and tumor cells in mixed samples are more closely associated than in separate samples (G). Error bars represent SEM, multiple slices of lung from n=3-4 biological replicates were analyzed with unpaired T-tests (H-I).

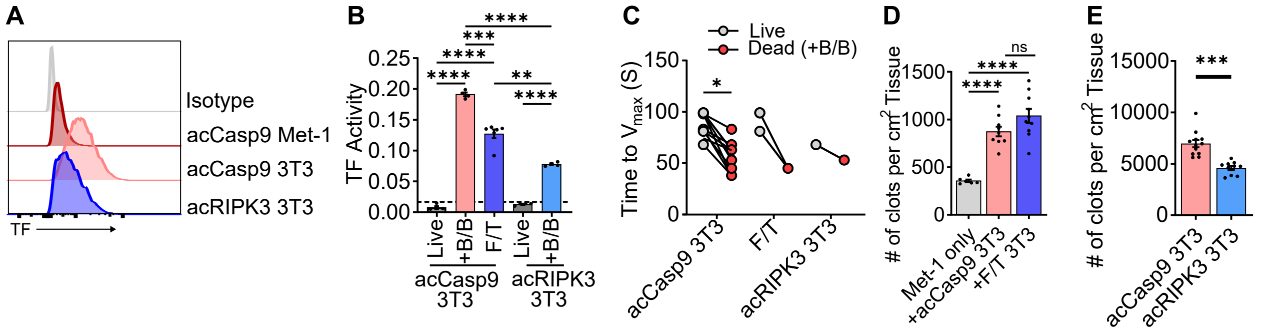

Supplementary Figure 8. Pro-coagulant activity of necrotic cells. TF-PE fluorescent intensity was measured by flow cytometry (A). TF activity was measured as the absorbance at 30 minutes after addition of live or apoptotic cells to mouse FVII, human FX, and chromogenic FXa substrate (B). Time to fibrin clot formation was measured as the maximum change in absorbance (Time to V_{max}) after adding Ca^{2+} to citrated mouse plasma, lower values indicate more rapid clot formation (C). Lungs were harvested at 15 min after I.V. and processed for fluorescent imaging (D-E). Statistical analysis is paired T-tests (C), Welch's ANOVA with Dunnett's multiple comparisons test (B,D) or unpaired t-test (E).

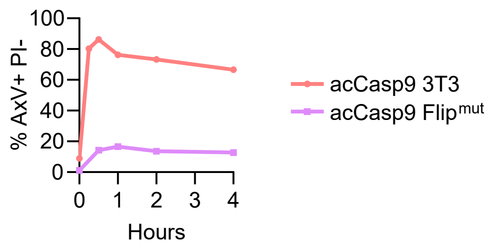

Supplementary Figure 9. Validation of Flip^{mut} cell line. Cells were activated with B/B and analyzed by flow cytometry at various timepoints, staining for PS exposure with Annexin V (AxV) and membrane integrity with propidium iodide (PI).

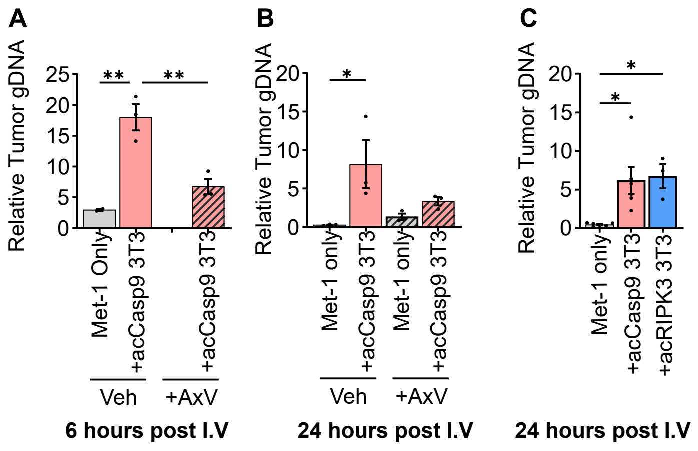

Supplementary Figure 10. qPCR quantification of tumor cell persistence in lungs. Lungs were harvested at indicated time points and tumor cell gDNA (PyMT) was isolated and quantified by qPCR. Statistical testing is Ordinary one-way ANOVA with Tu key's multiple comparisons test.

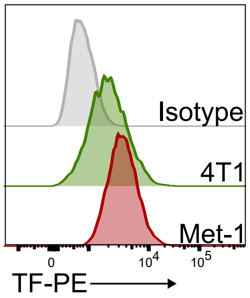

Supplementary Figure 11. TF expression of 4T1 cells. TF-PE fluorescent intensity was measured by flow cytometry.

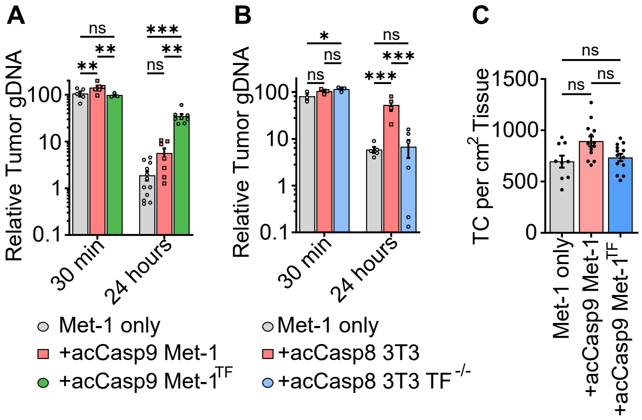

Supplementary Figure 12. Quantification of tumor cell seeding and persistence. Lungs were harvested at indicated time points and tumor cell gDNA (PyMT) was isolated and quantified by qPCR (A-B) or lungs were stained for immunofluorescent microscopy. Statistical testing is two-way ANOVA with Tu key's multiple comparisons test.

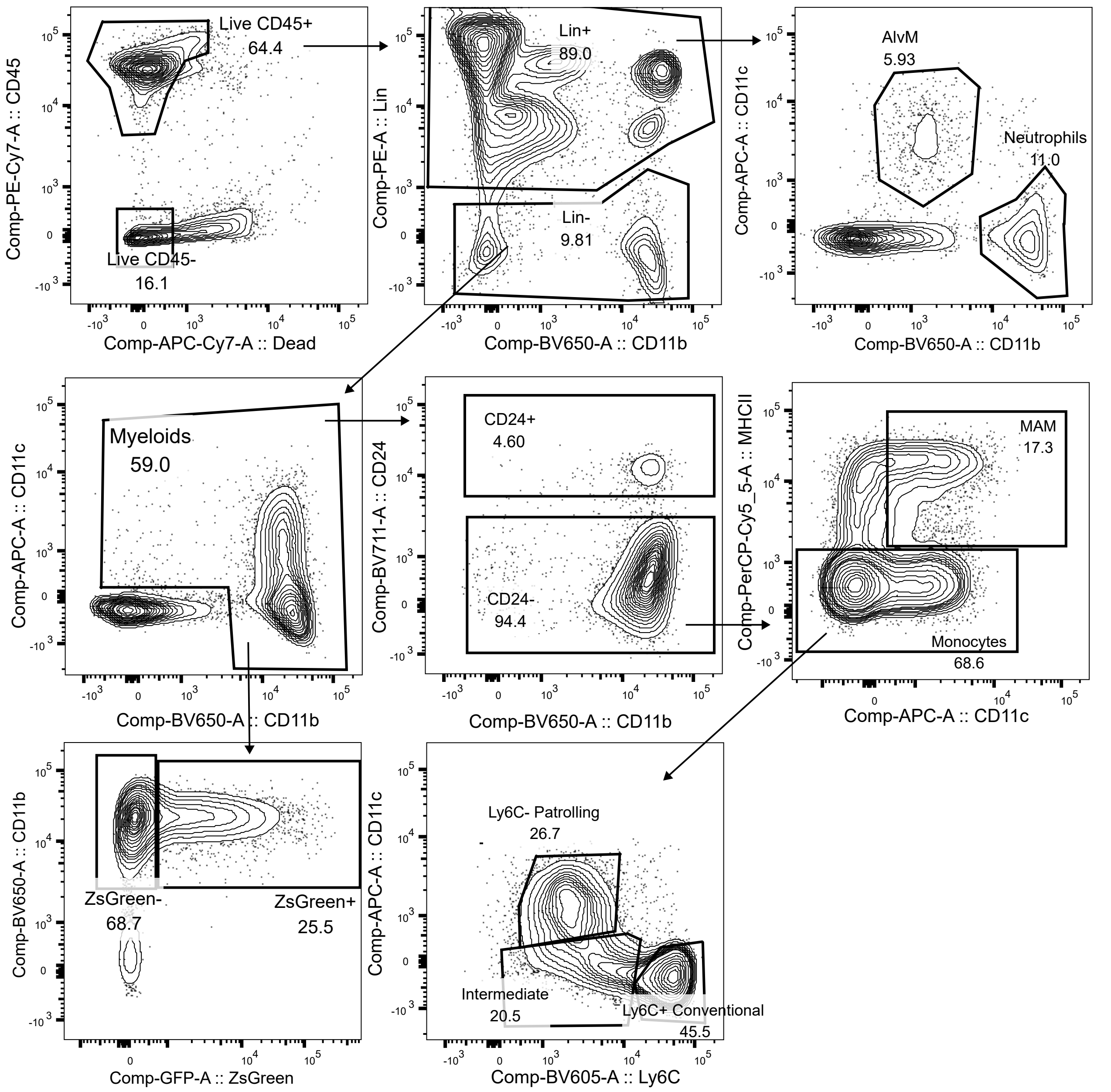

Supplementary Figure 13. Lung Myeloid Gating Scheme. Lungs were harvested from mice at 24 hours following I.V. injection of ZsGreen expressing cells. Cells were first gated using FSC and SSC, then singlets gating before gating on myeloid populations as indicated.

Target	Fwd Primer	Rev Primer	FAM/NFQ-MGB Probe
**Ptger2**	TAC CTT CAG CTG TAC GCC AC	GCC AGG AGA ATG AGG TGG TC	/56-FAM/CC TGC TGC T/ZEN/T ATC GTG GCT G/3IABkFQ/
**ZsGreen**	GTA CCA CGA GTC CAA GTT CTA C	CAC GTC GCC CTT CAA GAT	/56-FAM/CC CGT GAT G/ZEN/A AGA AGA TGA CCG ACA A/3IABkFQ/
**PyMT**	CGA AAT CCT TGT GTT GCT GA	GCT GGT CTT GGT CGC TTT C	/56-FAM/CC GAT GAC A/ZEN/G CAT ATC CCC /3IABkFQ/
**mCherry**	GAC TAC TTG AAG CTG TCC TTC C	CGC AGC TTC ACC TTG TAG AT	/56-FAM/TT CAA GTG G/ZEN/G AGC GCG TGA TGA A/3IABkFQ/
**GFP**	GAA CCG CAT CGA GCT GAA	TGC TTG TCG GCC ATG ATA TAG	/56-FAM/AT CGA CTT C/ZEN/A AGG AGG ACG GCA AC/3IABkFQ/
**101a**	GAG GAG ACT GTA CGC AAG ATG	TGG CGC TGC TGT TTG ATA	/56-FAM/AT CAC CA C C/ZEN/T TCA CCT CGT TGC C/3IABkFQ/

Supplementary Table 1. qPCR Primer/Probe Sets

**Antigen**	**Fluorophore**	**Clone**	**Manufacturer**	**Catalogue Number**	**Dilution**
CD45	PE-Cy7	30-F11	Biolegend	103114	1:100
Tissue Factor	PE	Polyclonal	R&D Systems	FAB3178P	1:100
CD3	PerCP-cy5.5	145-2C11	BD Biosciences	561108	1:100
NKp46	FITC	PK136	Biolegend	137606	1:100
MHCII	PerCP Cy5.5	M5/114.15.2	eBioscience	15-5321-82	1:400
Ly6G	PE	1A8	BD Biosciences	551461	1:500
SiglecF	PE	E50-2440	BD Pharmingen	562068	1:500
CD19	PE	1D3	BD Biosciences	557399	1:500
CD90.1	PE	OX-7	BD Biosciences	551401	1:500
NK1.1	PE	PK136	BD Biosciences	557391	1:100
CD11c	APC	N418	Biolegend	117310	1:100
CD24	BV711	M1/69	BD Biosciences	563450	1:400
CD11b	BV650	M1/70	Biolegend	101259	1:200
Ly6c	BV605	AL-21	BD Biosciences	563011	1:200

Supplementary Table 2. Flow Cytometry Antibody Information
