# Supplementary material for: Apoptotic cells promote circulating tumor cell survival and metastasis
Source: Commun Biol. 2025 Jul 29;8:1121. doi: 10.1038/s42003-025-08541-7 (PMC12307979; doi:10.1038/s42003-025-08541-7)
Supplement: Supplementary file 3 — Reporting Summary [file 42003_2025_8541_MOESM3_ESM.pdf]

nature portfolio

Corresponding author(s): Andrew Oberst

Last updated by author(s): Jun 24, 2025

Reporting Summary

Nature Portfolio wishes to improve the reproducibility of the work that we publish. This form provides structure for consistency and transparency in reporting. For further information on Nature Portfolio policies, see our Editorial Policies and the Editorial Policy Checklist.

Statistics

For all statistical analyses, confirm that the following items are present in the figure legend, table legend, main text, or Methods section.

n/a	Confirmed	
☐	☒	The exact sample size (*n*) for each experimental group/condition, given as a discrete number and unit of measurement
☐	☒	A statement on whether measurements were taken from distinct samples or whether the same sample was measured repeatedly
☐	☒	The statistical test(s) used AND whether they are one- or two-sided<br>*Only common tests should be described solely by name; describe more complex techniques in the Methods section.*
☒	☐	A description of all covariates tested
☐	☒	A description of any assumptions or corrections, such as tests of normality and adjustment for multiple comparisons
☐	☒	A full description of the statistical parameters including central tendency (e.g. means) or other basic estimates (e.g. regression coefficient) AND variation (e.g. standard deviation) or associated estimates of uncertainty (e.g. confidence intervals)
☐	☒	For null hypothesis testing, the test statistic (e.g. F, t, r) with confidence intervals, effect sizes, degrees of freedom and P value noted<br>*Give P values as exact values whenever suitable.*
☒	☐	For Bayesian analysis, information on the choice of priors and Markov chain Monte Carlo settings
☒	☐	For hierarchical and complex designs, identification of the appropriate level for tests and full reporting of outcomes
☒	☐	Estimates of effect sizes (e.g. Cohen's *d*, Pearson's *r*), indicating how they were calculated

Our web collection on statistics for biologists contains articles on many of the points above.

Software and code

Policy information about availability of computer code

Data collection	BD FACSDiva was used for collection of Flow Cytometry data, LASX Navigator was used for collecting microscope images, QuantStudio Real-Time PCR Software was used for collecting qPCR, Gen5 3.11 was used for spectrophotometry data collection
Data analysis	GraphPad Prism 10.2.3 was used for statistical testing and data visualization, ImageJ 1.54f was used for image quantification, QuPath 0.5.0 was used for spatial imaging analysis

For manuscripts utilizing custom algorithms or software that are central to the research but not yet described in published literature, software must be made available to editors and reviewers. We strongly encourage code deposition in a community repository (e.g. GitHub). See the Nature Portfolio guidelines for submitting code & software for further information.

Data

Policy information about availability of data

All manuscripts must include a data availability statement. This statement should provide the following information, where applicable:

- Accession codes, unique identifiers, or web links for publicly available datasets
- A description of any restrictions on data availability
- For clinical datasets or third party data, please ensure that the statement adheres to our policy

Data values underlying all figures can be found at Figshare under Hagan, Cassidy (2024). Supporting Data Values for "Apoptotic cells promote circulating tumor cell survival and metastasis". figshare. Dataset. https://doi.org/10.6084/m9.figshare.25872388.v1

Research involving human participants, their data, or biological material

Policy information about studies with human participants or human data. See also policy information about sex, gender (identity/presentation), and sexual orientation and race, ethnicity and racism.

Reporting on sex and gender	Research did not involve human participants
Reporting on race, ethnicity, or other socially relevant groupings	Research did not involve human participants
Population characteristics	Research did not involve human participants
Recruitment	Research did not involve human participants
Ethics oversight	Research did not involve human participants

Note that full information on the approval of the study protocol must also be provided in the manuscript.

Field-specific reporting

Please select the one below that is the best fit for your research. If you are not sure, read the appropriate sections before making your selection.

☒ Life sciences ☐ Behavioural & social sciences ☐ Ecological, evolutionary & environmental sciences

For a reference copy of the document with all sections, see nature.com/documents/nr-reporting-summary-flat.pdf

Life sciences study design

All studies must disclose on these points even when the disclosure is negative.

Sample size	Experimental means and standard deviation from preliminary experiments was used in a power calculation, with power set to 0.8 and type I error rate set to 5% to give an estimated required sample size.
Data exclusions	No data was excluded from analysis
Replication	All key results were replicated and confirmed in multiple independently performed experiments
Randomization	Mice were assigned randomly to groups
Blinding	Data was acquired in a blinded fashion until analysis was performed. The investigator performing lung metastasis counting did not know to which group samples belonged.

Reporting for specific materials, systems and methods

We require information from authors about some types of materials, experimental systems and methods used in many studies. Here, indicate whether each material, system or method listed is relevant to your study. If you are not sure if a list item applies to your research, read the appropriate section before selecting a response.

Materials & experimental systems

n/a	Involved in the study	
☐	☒	Antibodies
☐	☒	Eukaryotic cell lines
☒	☐	Palaeontology and archaeology
☐	☒	Animals and other organisms
☒	☐	Clinical data
☒	☐	Dual use research of concern
☒	☐	Plants

Methods

n/a	Involved in the study	
☒	☐	ChIP-seq
☐	☒	Flow cytometry
☒	☐	MRI-based neuroimaging

Antibodies

Antibodies used	anti-NK1.1 clone PK136 (Bio X Cell), Isotype control IgG2a clone C1.18.4 (Bio X Cell), CD45 clone 30-F11 (Biolegend), Tissue Factor polyclonal (R&D Systems), CD3 clone 145-2C11 (BD Bioscience), NKp46 clone PK136 (Biolegend), anti-GPIbβ conjugated to Dylight 649 (X649, Emfret analytics), rabbit anti-mCherry polyclonal (Rockland),MHCII clone M5/114.15.2 (eBioscience), Ly6G clone 1A8 (BD

Biosciences), SiglecF clone E50-2440 (BD Pharmingen), CD19 clone 1D3 (BD Biosciences), CD90.1 clone OX-7 (BD Biosciences), NK1.1 clone PK136 (BD Biosciences), CD11c clone N418 (BD Biosciences), CD24 clone M1/69 (BD Biosciences), CD11b clone M1/70 (BioLegend), Ly6C clone AL-21 (BD Biosciences),Rabbit anti-Cleaved Caspase-3 (Asp175, Cell Signaling Technology)

Validation	All antibodies used were purchased from reputable vendors that state validation methods on their website

Eukaryotic cell lines

Policy information about cell lines and Sex and Gender in Research

Cell line source(s)	Mouse Embryonic Fibroblasts (MEFs) were derived from day 15.5 embryos of B6/J or FVB/N pregnant mice. B16.F10 (CRL-6475) and NIH/3T3 (CRL-1658) cells were purchased from ATCC. Met-1 cells were derived from mammary carcinomas in FVB/N-Tg(MMTV-PyVmT) and kindly provided by Dr. Alexander Borowsky
Authentication	Cell lines were not authenticated but were continually observed and no indication of contamination was ever observed.
Mycoplasma contamination	Cell lines tested negative for Mycoplasma contamination throughout the duration of the study
Commonly misidentified lines (See ICLAC register)	No commonly misidentified cell lines were used

Animals and other research organisms

Policy information about studies involving animals; ARRIVE guidelines recommended for reporting animal research, and Sex and Gender in Research

Laboratory animals	C57BL6/J and FVB/NJ female mice from Jackson Laboratory were used between the ages of 6-10 weeks old
Wild animals	No wild animals were used
Reporting on sex	Our study examined only female animals. It is unknown whether the findings are relevant for male mice.
Field-collected samples	No field collected samples were used
Ethics oversight	All animals were maintained according to protocols approved by the University of Washington Institutional Animal Care and Use Committee (IACUC), under protocol number 4298-01 (PI: AO).

Note that full information on the approval of the study protocol must also be provided in the manuscript.

Plants

Seed stocks	No plants were used
Novel plant genotypes	No plants were used
Authentication	No plants were used

Flow Cytometry

Plots

Confirm that:

☒ The axis labels state the marker and fluorochrome used (e.g. CD4-FITC).

☒ The axis scales are clearly visible. Include numbers along axes only for bottom left plot of group (a 'group' is an analysis of identical markers).

☒ All plots are contour plots with outliers or pseudocolor plots.

☒ A numerical value for number of cells or percentage (with statistics) is provided.

Methodology

Sample preparation	Lungs were rinsed in PBS, roughly dissociated with scissors, then incubated with 50μg/mL Liberase TM (Sigma), 250μg/mL DNAse I (Sigma) in HBSS with Ca2+&Mg2+ for 35 minutes at 37C with gentle agitation. Tissue was homogenized with

GentleMACS lung dissociation (Miltenyi Biotec) and strained through 70µm cell strainers. Single cell suspensions were stained with fluorochrome conjugated antibodies in PBS with 3% FBS +0.05% NaAzide.

Instrument	CantoRUO or Symphony flow cytometer (BD Biosciences)
Software	Data was collected with FACSDiva (BD Biosciences) and analyzed with FlowJo software (TreeStar).
Cell population abundance	Cell population abundances of interest are quantified in the figures
Gating strategy	Gating strategy for all flow data is clearly outlined in figure legends and supplementary information

☒ Tick this box to confirm that a figure exemplifying the gating strategy is provided in the Supplementary Information.
