# Supplementary material for: Apoptotic cells promote circulating tumor cell survival and metastasis
Source: Commun Biol. 2025 Jul 29;8:1121. doi: 10.1038/s42003-025-08541-7 (PMC12307979; doi:10.1038/s42003-025-08541-7)
Supplement: Supplementary file 1 — Transparent Peer Review file [file 42003_2025_8541_MOESM1_ESM.pdf]

nature portfolio

Peer Review File

Apoptotic cells promote circulating tumor cell survival and metastasis

Corresponding Author: Dr Andrew Oberst

This file contains all reviewer reports in order by version, followed by all author rebuttals in order by version.

This manuscript has been previously reviewed at another journal. The peer review file only contains information relating to versions considered at Communications Biology.

Version 0:

Reviewer comments:

Reviewer #1

(Remarks to the Author)
I believe the novelty and scientific value presented in this manuscript align with the scope of this journal. Accept.

Open Access This Peer Review File is licensed under a Creative Commons Attribution 4.0 International License, which permits use, sharing, adaptation, distribution and reproduction in any medium or format, as long as you give appropriate credit to the original author(s) and the source, provide a link to the Creative Commons license, and indicate if changes were made.
In cases where reviewers are anonymous, credit should be given to 'Anonymous Referee' and the source.
The images or other third party material in this Peer Review File are included in the article's Creative Commons license, unless indicated otherwise in a credit line to the material. If material is not included in the article's Creative Commons license and your intended use is not permitted by statutory regulation or exceeds the permitted use, you will need to obtain permission directly from the copyright holder.
To view a copy of this license, visit https://creativecommons.org/licenses/by/4.0/
